# Open and strong-scaling tools for atom-probe crystallography: high-throughput methods for indexing crystal structure and orientation

**DOI:** 10.1107/S1600576721008578

**Published:** 2021-10-01

**Authors:** Markus Kühbach, Matthew Kasemer, Baptiste Gault, Andrew Breen

**Affiliations:** a Max-Planck-Institut für Eisenforschung GmbH, Max-Planck-Strasse 1, D-40237 Düsseldorf, Germany; b Fritz Haber Institute of the Max Planck Society, Faradayweg 4-6, D-14195 Berlin, Germany; c Department of Mechanical Engineering, University of Alabama, Tuscaloosa, AL 35487, USA; d Department of Materials, Imperial College London, Royal School of Mines, London, United Kingdom; e University of Sydney, Australian Centre for Microscopy and Microanalysis, NSW 2006 Sydney, Australia

**Keywords:** crystal structure identification, atom-probe tomography, orientation mapping, point cloud data, parallelization

## Abstract

Volumetric crystal structure indexing and orientation mapping are key data processing steps for virtually any quantitative study of spatial correlations between the local chemical composition features and the microstructure of a material. For electron and X-ray diffraction methods it is possible to develop indexing tools which compare measured and analytically computed patterns to decode the structure and relative orientation within local regions of interest. Consequently, a number of numerically efficient and automated software tools exist to solve the above characterization tasks. For atom-probe tomography (APT) experiments, however, the strategy of making comparisons between measured and analytically computed patterns is less robust because many APT data sets contain substantial noise. Given that sufficiently general predictive models for such noise remain elusive, crystallography tools for APT face several limitations: their robustness to noise is limited, and therefore so too is their capability to identify and distinguish different crystal structures and orientations. In addition, the tools are sequential and demand substantial manual interaction. In combination, this makes robust uncertainty quantification with automated high-throughput studies of the latent crystallographic information a difficult task with APT data. To improve the situation, the existing methods are reviewed and how they link to the methods currently used by the electron and X-ray diffraction communities is discussed. As a result of this, some of the APT methods are modified to yield more robust descriptors of the atomic arrangement. Also reported is how this enables the development of an open-source software tool for strong scaling and automated identification of a crystal structure, and the mapping of crystal orientation in nanocrystalline APT data sets with multiple phases.

## Abstract 2

It is now recognized that, beyond composition mapping in three dimensions, atom-probe tomography can provide partial crystallographic information for the material volume under investigation. Detailed here is an original solution for identifying the crystal structure and crystal orientations within atom-probe tomographic reconstructions. The reported tools are open-source software and scale strongly on at least 3200 computing cores or 160 graphics cards.

## 1. Introduction   

Atom-probe tomography (APT) and the related technique of field-ion microscopy are powerful nanoscale analytical tools capable of reconstructing the 3D position and chemical identity of millions of individual atoms from a specimen (Müller *et al.*, 1968 ▸; Blavette *et al.*, 1993 ▸; Miller, 2000 ▸; Gault *et al.*, 2012*c* ▸; Larson *et al.*, 2013*a* ▸,*b* ▸; Lefebvre *et al.*, 2016 ▸) with sub-nanometre resolution (Kelly *et al.*, 2007 ▸, 2009 ▸; Gault *et al.*, 2009*b* ▸, 2010*b* ▸; De Geuser & Gault, 2020 ▸). This unique capability makes APT a useful technique to study the 3D atomic architecture of solids and has provided invaluable scientific insight into fields such as materials science (Hono, 1999 ▸; Herbig *et al.*, 2014 ▸; Kuzmina *et al.*, 2015 ▸; Chen *et al.*, 2017 ▸), geology (Valley *et al.*, 2014 ▸; Piazolo *et al.*, 2016 ▸; Saxey *et al.*, 2018 ▸), semiconductors (Castell *et al.*, 2003 ▸; Giddings *et al.*, 2018 ▸; Rigutti *et al.*, 2018 ▸) and even biology (Gordon & Joester, 2011 ▸).

The technique works by inserting a sharp needle-shaped specimen with an end-tip radius of less than 100 nm into an ultra-high-vacuum chamber (<1.4 × 10^−13^ bar; 1 bar = 100 000 Pa). A standing voltage of a few kilovolts is applied, on top of which either laser or high-voltage pulses are superimposed to induce time-controlled field evaporation of individual atoms from the surface. During these experiments the specimen is held at cryogenic temperatures in the range of 25–80 K to limit the influence of surface diffusion on the analyses.

Accelerated by the electric field surrounding the specimen, the ions are collected by a position-sensitive and time-resolved detector. Information on the time of flight between the specimen and the detector enables the determination of the mass-to-charge ratio for each ion. These ratios are associated with the most likely elemental identity, *i.e.* the atom type of each ion (Hudson *et al.*, 2011 ▸; Haley *et al.*, 2017 ▸). A reverse-projection algorithm, which uses the sequence of evaporation events, combined with the *x* and *y* detector hit positions of the ions, is used to reconstruct the 3D position of the atoms from the analysed specimen (Bas *et al.*, 1995 ▸; Geiser *et al.*, 2009 ▸; Gault *et al.*, 2010*a* ▸, 2011 ▸; Hatzeglou & Vurpillot, 2019 ▸; Fletcher *et al.*, 2020 ▸). The result is a 3D data set of atom positions and associated (molecular) ion types with which one can study the atom(s) at reconstructed positions. Ultimately, crystallographic information may be determined from such data, known by the term atom-probe crystallography in recent literature (Moody *et al.*, 2011 ▸; Araullo-Peters *et al.*, 2012 ▸; Gault *et al.*, 2012*b* ▸).

APT data sets of crystalline materials often contain crystallographic information that is particularly useful for the calibration of the tomographic reconstruction (Gault *et al.*, 2008 ▸, 2009*a* ▸), the measurement of the crystallographic character of the interfaces (Breen *et al.*, 2017 ▸) and the study of ordering (Marquis *et al.*, 2007 ▸; Gault *et al.*, 2012*a* ▸; Bagot *et al.*, 2017 ▸). However, the crystallographic information is partially lost because of the limited spatial resolution, finite detection efficiency and necessary simplifying assumptions made when applying back-projection algorithms (Vurpillot *et al.*, 2000 ▸; Kelly *et al.*, 2007 ▸; Gault *et al.*, 2009*b* ▸, 2010*b* ▸, 2021 ▸; Vurpillot & Oberdorfer, 2015 ▸; Jenkins *et al.*, 2020 ▸; De Geuser & Gault, 2020 ▸). In effect, these limitations result in noise which makes the retrieval and exploitation of crystallographic information with APT data sets more difficult than if one were to infer structural information from data- or time-averaged molecular dynamics or diffraction methods.

The experimental conditions and the physical properties of the material being analysed affect the available crystallographic detail. Typically, pure metals such as aluminium and tungsten collected at low temperature contain the clearest crystallographic information (Gault *et al.*, 2010*b* ▸,*c* ▸). For more complex materials systems, this information becomes more difficult to observe, in particular when different phases give rise to locally varying field evaporation conditions (Vurpillot *et al.*, 2000 ▸). Increasing the base temperature of the analysis or using a laser-pulsing mode to improve specimen yield has a detrimental effect on the quality of the crystallographic information that can be retrieved (Gault *et al.*, 2010*b* ▸,*c* ▸). This substantiates the need for robust methods and efficient implementations to give practitioners a tool for quantifying where a data set contains accurate crystallographic information.

A working strategy for extracting crystallographic information from data sets of single- and polycrystalline specimens is to evaluate the pattern in the hit densities in detector space. Such patterns are characterized by averaging the number of subsequent *x* and *y* detector hit positions from a few hundred thousand to a few million ions into a hit density pattern (Moody *et al.*, 2011 ▸; Yao, 2016 ▸; Wei *et al.*, 2018 ▸, 2019 ▸; Kühbach *et al.*, 2019*b* ▸). Such patterns form as a result of trajectory aberrations inherently related to the crystallography of the specimen and quantum effects (Oberdorfer *et al.*, 2013 ▸; Ashton *et al.*, 2020 ▸). The necessity of collecting these patterns via integrating the signal over a substantial number of ions (through averaging over the entire effective detector area as well as with depth) makes this strategy spatially inaccurate. In addition, the detector space does not account for eventual spatial distortions along the main axis of the data set. In effect, it is difficult in practice to index different thermodynamic phases via differences in their crystal structures or to extract more information on the shape of these phases or the (relative) orientations of their lattices.

By contrast, this is the strength of electron diffraction methods. In particular, correlative electron diffraction methods performed on a specimen prior to running an atom-probe experiment, such as transmission Kikuchi diffraction (TKD) (Babinsky *et al.*, 2014 ▸; Zaefferer, 2011 ▸; Keller & Geiss, 2011 ▸; Trimby, 2012 ▸; Trimby *et al.*, 2014 ▸; Sneddon *et al.*, 2016 ▸; Breen *et al.*, 2017 ▸; Schwarz *et al.*, 2017 ▸) and transmission electron microscopy (TEM) techniques (Herbig, 2018 ▸), such as nanobeam diffraction (Herbig *et al.*, 2014 ▸; Zhou *et al.*, 2016 ▸) or high-resolution TEM (Makineni *et al.*, 2018 ▸; Liebscher *et al.*, 2018 ▸) methods like precession electron diffraction (Midgley & Eggeman, 2015 ▸), are particularly useful in providing some crystallographic information, including crystal defects within the grains and at the grain or phase boundaries. Applying these correlative techniques, however, adds to further experimental complexity and presents challenges with respect to the alignment between the electron microscopy and APT data (Mouton *et al.*, 2019 ▸).

Another strategy for reconstructing grain and phase boundaries implicitly within APT data sets is via analysing regions of preferred elemental segregation. In fact, when different atom types can serve as markers, gradients in the nanoscale composition can be detected via segmentation methods like isosurfaces (Hellman *et al.*, 1999 ▸) or an extraction of cell facets from tessellations (Felfer *et al.*, 2012 ▸, 2013 ▸), or via artificial intelligence methods (Zhou *et al.*, 2021 ▸). Without analysing the specific atomic arrangement at the interface, however, deducing the orientations of the adjoining crystals is an ill-posed task.

All the above arguments affirm the advantages of direct crystallographic measurements on the reconstructed atom positions within APT data sets. In many cases, latent crystallographic information is contained in such data sets but it is incomplete. At present, the lack of simple and efficient crystallographic processing tools means that such data go largely underused. Given that crystallographic information is often incomplete, it is essential to quantify at which locations in the data set it is available, how accurate and precise it is, and where such crystallographic information is virtually not re­coverable. Assessments of the same data set with several of the above-described crystallographic methods, *i.e.* heads-up, can help to clarify when analysis of the reconstructed atom positions is substantiated, or when correlative microscopy, elemental segregation or patterns in the detector space are the last resort.

The above analysis tasks do not appreciably differ conceptually or fundamentally from the reconstruction of grains from atomic positions monitored in molecular dynamic (MD) simulations (Stukowski, 2012 ▸; Larsen *et al.*, 2016 ▸; Hoffrogge & Barrales-Mora, 2017 ▸). However, APT data sets display, in most cases, a stronger positional noise than is shown in MD simulations or expected from thermal lattice vibrations alone (Lonsdale, 1948 ▸; Vurpillot *et al.*, 2000 ▸). In addition to thermal lattice vibrations and eventual diffusion over the specimen surface prior to launch, reconstructed APT data sets inherit inaccuracies such as missing atoms (10–30%) because of limited detector efficiency.

This explains the motivation in the past for developing specific approaches for APT data to mitigate the above challenges (Vurpillot *et al.*, 2003 ▸; Moody *et al.*, 2011 ▸, 2014 ▸; Breen *et al.*, 2015 ▸; Wallace *et al.*, 2018 ▸). Despite their success for single crystals, though, the methods and their implementation in software tools present practical limitations that justify further research on highly localized 3D orientation mapping methods. One limitation of the tools when applying them for routine characterization of volumetric data is a lack of support for parallel processing. Another limitation is connected to the different conceptual design of these earlier software tools which builds on using a GUI interface to perform manual data analyses, whereas our approach is to develop a complementary tool for performing automated high-throughput studies.

In addition, we observe that the landscape with respect to numerical methods and computational hardware has improved substantially over the past decade, bringing a new opportunity for computationally intensive structural and crystallographic analysis (Favre-Nicolin *et al.*, 2011 ▸; Katnagallu *et al.*, 2017 ▸; Di Bernardo, 2018 ▸; Kühbach *et al.*, 2021 ▸) that was not practical in the earlier work on 3D atom-probe crystallography (Camus *et al.*, 1995 ▸; Cerezo *et al.*, 1998 ▸; Vurpillot *et al.*, 2001 ▸). Many- and multi-core central processing units (CPUs) and general purpose graphics card coprocessors (GPGPUs, accelerators, or GPUs for short) are now readily available. This has made orders of magnitude more processing power available to microscopists and microanalysts. These observations motivate this study.

Thus, we aim to close several gaps in atom-probe crystallography. First, we generalize existing atom-probe crystallography methods to make these tools more robust for indexing arbitrary crystal structures. Next, we detail how these results can be used to develop an automated method for detecting specific crystal structures within APT reconstructions and indexing their respective crystallographic orientations. We analyse specifically the robustness against positional noise and missing atoms. Next, synthetic data sets with a low or high complexity of the grain- and phase-boundary network will be assessed to verify the methods. Thereafter, we assess application examples on experimental APT data sets to identify the capabilities and quantify the limitations of such methods. Finally, we show how this work fits into the larger picture of indexing crystal structure and mapping orientation via the existing methods of the electron and X-ray diffraction communities (Campbell, 1998 ▸; Kolb *et al.*, 2007 ▸; Maia *et al.*, 2011 ▸; Lenthe *et al.*, 2019 ▸; Hielscher *et al.*, 2019 ▸). We implement the numerical tools for the above research as open-source software with specific parallelized algorithms (for CPUs and GPUs). In effect, our work closes several gaps by delivering a tool for reusable, reliable and orders of magnitude faster high-throughput methods for atom-probe crystallography.

## 2. Computational methods   

### 2.1. General procedure for indexing crystal structure and orientation   

We perform all analyses by scanning the data-set volume with a nanometre-sized (spherical) region of interest (ROI). This yields a collection of ROIs. Each local analysis for an ROI has two steps. In the first step, at least one (crystallographic) signature is computed from the positions of selected atom types in the ROI. A signature encodes when there is a long-range periodic arrangement of selected atom types along particular (crystallographic) directions. Multiple signatures (for different atom types) are computed per ROI and evaluated as a set of signatures to help distinguish different crystal structures. The signatures are images whose formatting is a function of the signature detection methods. In the second step, we use the signature(s) for each ROI to index the most likely matching crystal structure, or candidate for short, and output the equivalent rotations that bring the encoded crystallographic directions in the signatures into a consistent alignment with the laboratory coordinate system.

Signatures are computed with two established, but here modified, methods from atom-probe crystallography, either the method of Araullo-Peters *et al.* (2015 ▸) or the method of Vurpillot *et al.* (2001 ▸). The discussion in this paper is focused on the first method. The novelty is not only in the fact that we modified this method to achieve more robust signatures than those computed in the original paper but also that we detail, for the first time for APT, how to output orientations systematically with these signatures. According to the reference space in which the two methods operate, we refer to the first method as the real-space method (RSP) and the second as the reciprocal-space method (FSP).

### 2.2. Methods for detecting signatures of long-range periodic atomic arrangements   

#### 2.2.1. Real-space method   

**Figure 1:**
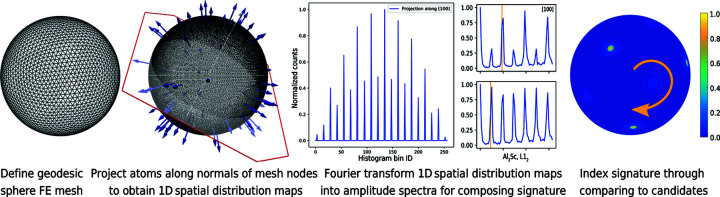
In the real-space method we compute signatures for each ROI and reference signatures of crystal structure candidates to compare these for indexing. The computation has several steps. First, the definition of one geodesic mesh which is used for all ROIs and defines the corresponding projection directions. Second, the computation of the 1D SDMs for each direction and ROI. Third, the fast Fourier transformation of each SDM plus subsequent signal extraction using the modified strategy for extracting crystal-structure-specific peaks from the amplitude spectra to compose at least one signature per ROI. Fourth, the indexing of the signatures by comparing each signature against a set of rotated references (signatures for crystal structure candidates). The colour bar to the right shows normalized intensities.

Beginning with the work of Araullo-Peters *et al.* (2015 ▸), we project interatom distances along a set of directions defined *a priori* (Araullo-Peters *et al.*, 2015 ▸; Haley *et al.*, 2019 ▸). The key steps are shown in Fig. 1 ▸. Each projection yields a 1D spatial distribution map (SDM) (Geiser *et al.*, 2007 ▸; Moody *et al.*, 2009*a* ▸,*b* ▸) and hence a set of histograms of projected distances for each ROI. A right-handed Cartesian coordinate system (laboratory) is assumed whose *xy* plane is located at the base of the data set. Each local analysis of an ROI yields a set of (projection) directions, encoded as elevation–azimuth pairs. We propose to align these directions with the outer unit normals of the nodes of a geodesic sphere finite-element mesh (Popko, 2012 ▸; Pokhrel *et al.*, 2018 ▸). In this work, the finite-element (FE) mesh contained *N*_v_ = 40 962 vertices. For each direction, *i.e.* 1D SDM, we compute one histogram using equation (1), 
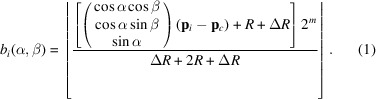
Here, *R* is the radius of each ROI, while α and β denote the elevation and azimuth, respectively. The histograms have a total of 2^*m*^ bins, with *m* an integer in the range from 8 to 12 and Δ*R* the bin width. Lower-case bold letters specify vectors in three dimensions. For each *c*th ROI, the position **p**_*i*_ of the *i*th atom is evaluated relative to the centre of the ROI **p**_*c*_. Projected distances are cast into a bin labelled *b*_*i*_.

Next, we compute a fast Fourier transformation (FFT) of each histogram and inspect the resulting amplitude spectrum for each direction. Provided that the lattice planes are aligned nearly perpendicular to a particular (projection) direction **v**, we expect to find peaks in the amplitude spectrum. These peaks should correlate with the spacing of particular distances between lattice planes or multiples of these distances. Consequently, a spherical image can be composed in such a way that it is a signature of the corresponding lattice periodicity signal for each direction within the ROI. By virtue of construction, each image also encodes the relative orientation of the crystal volume.

The key modification to the original work (Araullo-Peters *et al.*, 2015 ▸) is in the choosing of particular bins instead of arbitrary bins of the amplitude spectrum to compose the signature. Repeating this choice for all directions results in a measured spherical image 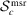 for each ROI *c*. When computed from a data set that we wish to index, such signatures are here referred to as measured signatures in order to distinguish them from the reference signatures that we explain in the following subsections.

The key advantage of this approach is that the computation can be executed independently for each direction and each ROI. This independence brings substantial potential for analysing the data using parallel computing. To the best of our knowledge no implementation has exploited this advantage so far. A detailed analysis of the numerical costs of the RSP method is reported in the supporting information.

#### 2.2.2. Reciprocal-space method   

Following Vurpillot *et al.* (2001 ▸), we implemented a second method for computing signatures that evaluates equation (2) to compute a direct Fourier transform of the positions for all atoms *N*_*w*_ of a selected atom type within each ROI *c*. Here, *w* is a counting variable, *i* is the imaginary unit and **k** is a reciprocal-space position vector: 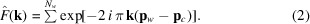
Contrary to signatures for an ROI obtained with RSP, *i.e.* spherical images, the reciprocal-space method yields the signatures as 3D image stacks. These image stacks probe a discretized subspace of reciprocal-space positions **k** ∈ [−2π, 2π]^3^. The reciprocal-space method also allows for substantial parallel computing, which we detail in the supporting information. However, a key difference from the RSP method is that this requires more arithmetic operations per ROI because there are typically far more reciprocal-space grid points (**k**) to compute than directions (*N*_v_). This has substantially restricted the application of the reciprocal-space method until recently (Katnagallu *et al.*, 2017 ▸; Di Bernardo, 2018 ▸) when the specific performance of GPUs became higher and their costs lower.

### 2.3. Extraction of the crystal structure and orientation   

Both RSP and FSP yield signatures of crystallographic information for each ROI. At least two strategies now exist to identify the crystal structure and orientation from these signatures: either we restrict ourselves to a particular small set of candidate crystal structures (candidates, for short) or we do not make such a simplification and try to test against all possible crystal structures. Here, we detail a solution in accordance with the first strategy.

Thus, it suffices to compare, for each ROI, each signature against a set of reference signatures for all candidates and rotated versions of the reference signatures. The strategy is similar to the indexing of electron backscatter diffraction (EBSD) data (Schwartz *et al.*, 2010 ▸) whereby a measured Kikuchi pattern is indexed with a predicted Kikuchi pattern while assuming a few different crystal structures as candidates. The best match is then quantified via suitable descriptors (Wright *et al.*, 2015 ▸).

Likewise, here we compare, for each ROI, the collected signatures against the reference signatures and rotated versions of these for a set of crystal structure candidates. The reference signatures for the candidates, or reference(s) for short, are characterized for synthetic single crystals with a defined atomic arrangement and defined noise. Specifically, the references are computed as atom-type-specific spherical images (for RSP) from synthetic single-crystal data sets. The resulting spherical images are rotated to sample the orientation space (Bunge, 1982 ▸; Rowenhorst *et al.*, 2015 ▸). Specifically, we computed the reference signatures for a discretized orientation set 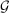 [probing the SO(3)] which contained approximately 620 000 orientations (also referred to as test orientations) with 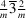 crystal symmetry and 1° angular spacing using the *MTEX* texture toolbox for MATLAB (Bachmann *et al.*, 2010*b* ▸). By virtue of construction, a measured signature encodes a particular variant of the possible symmetric variants for an orientation. Therefore, we include the variants in the above set. Further details are reported in the supporting information.

### 2.4. Indexing   

With the above assumptions and definitions, the task of indexing the crystal structure and orientation reduces for each ROI and crystal structure candidate to a comparison of at least one spherical image (in the case of RSP), *i.e.* signature, with a set of rotated spherical images, *i.e.* rotated references (signatures). To accomplish this, one can either evaluate the image intensities as a whole, *e.g.* via cross correlation, or register the images by matching against spots of high image intensity.

The indexing algorithm works as follows. First, we normalize the image intensities of the signatures for the ROIs and for the references for each candidate. Second, we identify the locations and intensities of a predefined number of the highest absolute image intensities for each candidate. Third, we build a lookup table which guides where to probe nodal values of the signature to compare implicitly against all rotated references for the orientation set 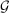. With this, we evaluate the signatures for each candidate and ROI at the precomputed image positions to quantify how closely the signatures and references match. Further numerical details and the costs of this approach are described in the supporting information.

### 2.5. Implementing these numerical methods into a software toolbox   

#### 2.5.1. Defining a workflow for indexing crystal structure and orientation   

We implemented the above methods as additional tools (*paraprobe-araullo* for the RSP and *para­probe-fourier* for the FSP method, plus *paraprobe-indexer*) in *PARAPROBE*. This software is an open-source toolbox for high-throughput analysis of APT data sets (Kühbach *et al.*, 2019*a* ▸, 2021 ▸). The ROIs were either placed on the positions of a 3D grid or placed via random sampling. Specific methods (Kühbach *et al.*, 2021 ▸) ensured that only ROIs within the data set were analysed. Furthermore, we implemented a proof of concept to an iterative grid refinement to allow detailed analyses at a much finer spatial resolution of the ROI grid without having to waste computational resources on locations where the signal quality is lower. The analysis workflow is defined through a Python script, the details of which are described in the supporting information. Hands-on examples in the form of Jupyter notebooks are provided to guide experimentalists to apply the methods to their own data sets (http://gitlab.com/paraprobe/paraprobe-toolbox.git). As we detail in Appendix *A* [App appa], the interested reader is referred to the newer version of the tool because it offers a simpler workflow.

#### 2.5.2. Parallel implementation of the software   

With several multi-core CPUs and GPUs typically on board, modern computers offer multiple layers of parallel resources (Hennessy & Patterson, 2012 ▸; Rauber & Rünger, 2013 ▸). To use all these resources productively, we built on previous work (Kühbach *et al.*, 2021 ▸) and parallelized for CPUs (real-space method) and also GPUs (reciprocal-space method). Using the Message Passing Interface (MPI) library, the ROIs were split into subsets. These subsets were delegated in a round-robin fashion to the computing nodes and distributed further on these via multithreading, realized with Open Multi-Processing (OpenMP) (Chapman *et al.*, 2007 ▸; Kühbach *et al.*, 2021 ▸). Atom positions were always queried using OpenMP. GPU instructions were implemented through Open Accelerator (OpenACC) compiler directives and Compute Unified Device Architecture (CUDA) library commands (Nvidia Corporation, 2019 ▸), respectively. Each GPU was instructed by its own MPI process and OpenMP master thread.

The Hierarchical Data Format (HDF5) library was used (The HDF Group, 2020 ▸; Prabhat, 2014 ▸) to store all data and metadata transparently and performantly. Herewith, we signify our desire to remove unnecessary barriers with respect to the FAIR data stewardship principles (Wilkinson *et al.*, 2016 ▸; Draxl & Scheffler, 2020 ▸). Specific sequential implementation tricks are detailed in the supporting information.

All analyses were executed on the TALOS computer cluster (Kühbach *et al.*, 2019*a* ▸, 2021 ▸; Kühbach & Roters, 2020 ▸). Each node has two Intel Xeon Gold 6138 20-core processors with access to 188 GiB main memory in total. Each node is equipped with two Nvidia Tesla Volta V100 (Nvidia Corporation, 2017 ▸) GPUs with 32 GB memory each. We used at most 80 of the TALOS computing nodes and their GPU pairs. All resources were used exclusively and the elapsed time for accessing files and processing data was accounted for individually. Further details are reported in the supporting information.

## 3. Results   

### 3.1. Verification of the methods   

#### 3.1.1. Does the real-space method yield signatures specific for a crystal structure?   

First, we verify that RSP yields peaks in the amplitude spectra at positions that are specific for the crystal structure and the orientation of the crystal. This is a requirement for reliably distinguishing between different crystal structures. Synthetic data sets were created for this purpose as these ensure a rigorously controlled atomic architecture. Specifically, we synthesized three cylindrical data sets with a height-to-radius ratio of *H* = 2*R* and a total of 25 × 10^6^ atoms:

(i) A face-centred cubic (f.c.c.) aluminium structure with *a*_Al_ = 4.05 Å [Crystallography Open Database (COD; Downs & Hall-Wallace, 2003 ▸; Gražulis *et al.*, 2009 ▸) ID 9008460].

(ii) An L1_2_ structure Al_3_Sc phase with *a*_Sc_ = 4.10 Å according to Villars & Cenzual (2016*b* ▸).

(iii) A body-centred cubic (b.c.c.) tungsten structure with *a*_W_ = 3.16 Å (COD ID 9008558).

All data sets represent single crystals (defect free and of single phase). Two instances were created for each of the three data sets. For the first instance the lattice remained unrotated, thereby representing a single crystal in (φ_1_ = 0.0°, Φ = 0.0°, φ_2_ = 0.0°) orientation using the Bunge–Euler notation. For the second instance, the single crystal was rotated (φ_1_ = 8.0°, Φ = 8.0°, φ_2_ = 8.0°).

First we work with the single crystals with unrotated lattice. We placed a single ROI (*R* = 20 Å, *m* = 8) in the centre of the data set and projected the atoms in the ROI along the 26 crystallographic variants of the 〈100〉, 〈110〉 and 〈111〉 crystallographic directions.

**Figure 2:**
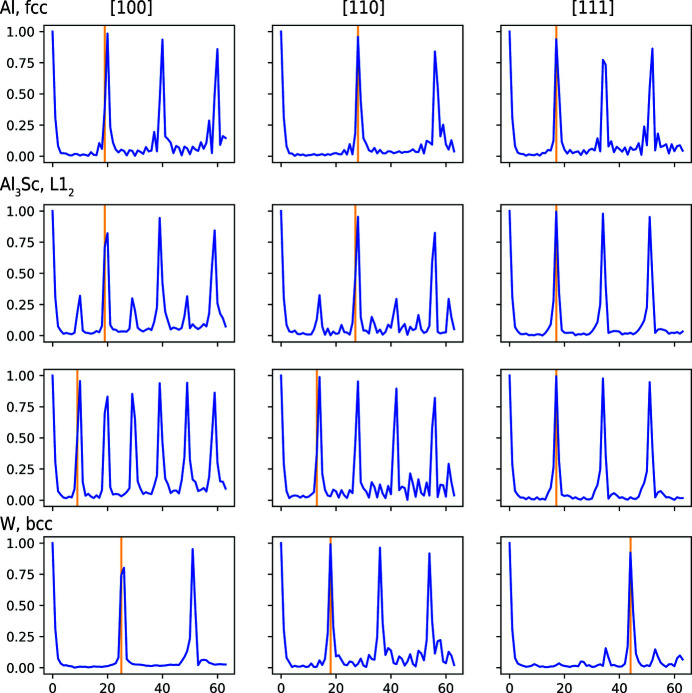
Verification that signatures can distinguish different crystal structures and encode different orientations. Compared are the low-frequency parts of the one-sided FFT amplitude spectra for the aluminium, Al_3_Sc and tungsten synthetic data sets after projecting along specific crystallographic directions ([110], [110] and [111]). The two rows in the middle display the results for the L1_2_ crystal structure (with the aluminium substructure in the upper and the scandium substructure in the lower middle row). The *x* axis shows amplitude spectrum bin IDs. The vertical orange lines mark the theoretical lattice plane spacing for lattice planes which are stacked perpendicular to the respective crystallographic (projection) directions. Exemplified for the b.c.c. tungsten structure, we expect to find an alternating sequence of 〈100〉 and 〈200〉 planes with 0.5*a*_W_ spacing and an equal planar density of the tungsten atoms for the two inspected crystallographic plane sets. We assume the signal length is 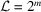 with *m* = 8. The sampling frequency is 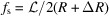 with Δ*R* = *R*/2^*m*−1^ − 1. For *R* = 2.0 nm and a reciprocal spacing *f* = 1/0.5*a*_W_, we can verify that the amplitude peaks in the 19th bin (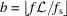).

Fig. 2 ▸ summarizes the results by comparing selected one-sided amplitude spectra of the fast-Fourier-transformed SDMs. Specifically, we compare amplitude spectra for all three crystal structures (rows of the image matrix) and selected specific crystallographic directions (columns of the image matrix). The results are representative for other analysed ROI radii and frequency resolutions. The figure demonstrates that the modified signal selection strategy yields amplitude spectra with distinct peaks. There is always a peak at the origin, which accounts for the total number of atoms for the analysed type in the ROI. Further peaks in different bins are detected. Their bin position encodes the spacing of a stack of lattice planes. As confirmed by the vertical orange lines, explained in further detail in the figure caption, the positions of the peaks match theoretical expectations.

When comparing the peaks for aluminium versus tungsten in the amplitude spectra for the [100] direction, for instance, it is reassuring to find that the peaks are in different bins because the lattice constant of aluminium is different from that of tungsten. The two rows in the middle of the figure depict how the peaks of the specific L1_2_ structure candidate differ from the signature for the aluminium structure, although the dimensions of both unit cells are almost the same.

We learn that to distinguish between these crystal structures, if they were to exist in the same data set, it is necessary to evaluate two amplitude spectra: one for the atoms of the aluminium substructure and another for the atoms of the scandium substructure. We also learn that observing a single peak in an amplitude spectrum characterizes the spacing between a specifically oriented single stack of lattice planes. In the examples above we have lattice planes with normals [±*u*00], with *u* as an integer.

Consequently, these peaks can be used to compose 3D signatures of the respective crystal structures which are specific for a given set of lattice planes including all symmetric variants {*hkl*}. For cubic crystal symmetry this implies that the signatures are capable of detecting equivalent 〈*uvw*〉 directions. It is possible to compose a signature from peaks at different locations and use a colouring scheme to distinguish the peaks.

In effect, the real-space method yields distinct signatures for a crystal structure. The Al_3_Sc case shows that if two structures have strong similarities, it is necessary to study a combination of signatures that are computed from different atom types and evaluate their corresponding sublattices.

Our procedure to specify the relevant bins in the amplitude spectra is more robust than the strategy of the original authors – see Section 2.2, bullet point 4(*c*) of the original paper (Araullo-Peters *et al.*, 2015 ▸). Therein, the authors proposed to select a peak at the bin which is closest to where the amplitude spectrum has its median amplitude. However, as we discussed, each bin represents a specific spacing. In effect, such a peak selection rule cannot guarantee that, for each projected direction and ROI, the same bins always get analysed.

#### 3.1.2. How robust are the signatures from RSP against noise?   

Before the computed signatures can be used for indexing, we need to address their robustness against noise because the reconstruction of an APT data set from detector-hit and time-of-flight measurements faces the challenges of undetected ions, trajectory aberrations and trajectory overlap (Larson *et al.*, 2013*a* ▸; Vurpillot & Oberdorfer, 2015 ▸; Devaraj *et al.*, 2018 ▸). Therefore, the effects of noise from finite detection efficiency, *i.e.* missing atoms in the reconstructed data set, and positional noise, *i.e.* imprecisely and/or inaccurately placed atoms, need to be accounted for. The spatial resolution within an atom-probe reconstruction is anisotropic and typically higher along the local normal to the specimen surface than in the local tangent plane. For this purpose, we proceed by building copies of the rotated aluminium synthetic data sets from the above verification. These data sets were modified to create two types of noisy copies:

(i) Atoms were kept at their position but partially removed in a spatially random manner so that *N*_*f*_ = η*N*_*i*_ atoms remain, with η = 1.0, 0.75, 0.50 and 0.25.

(ii) Atoms were not removed but displaced by applying an anisotropic Gaussian displacement kernel. The standard deviation of the kernel was always σ_*x*_ = σ_*y*_ = σ_*xy*_ = 2σ_*z*_ but different σ_*z*_ = 0.00, 0.25, 0.50, 0.75, 1.00 and 2.00 Å were probed.

**Figure 3:**
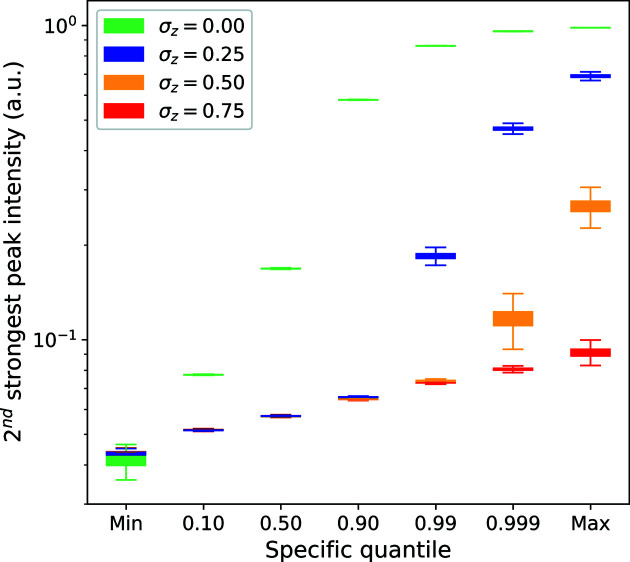
Quantification of the real-space method against positional noise. For each ROI, we identify the peaks in all of the amplitude spectra and report the individually second-strongest peak for each amplitude spectrum. With *N*_v_ FE mesh nodes, *i.e. N*_v_ directions (or corresponding SDMs), this yields one cumulative distribution per ROI. Next, specific quantiles of the distribution were extracted for each ROI and displayed for the entire ROI ensemble. This condenses how the results differ for all amplitude spectra (40 962 per ROI) and all ROIs (10 000 in total). We repeat this statistical analysis for all signatures from the data sets with different atom displacement strengths (σ_*z*_) and compare them. Contrary to noise from missing atoms, a substantial reduction in the signal-to-noise ratio is observed the more strongly the atoms are displaced.

In addition to contributions from noise, rigorous analyses also need to take into account that placing the ROIs in the data set is random sampling. In effect, we expect that the nodal intensities of the signatures scatter statistically, especially when using ROIs with a radius of just a few ångströms. To quantify such scatter, we work with a statistical ensemble of 1 × 10^4^ ROIs for each synthetic data set. The results are documented in Fig. 3 ▸ and in the supporting information.

The figures display descriptive statistics for selected quantiles of the distribution of the second-strongest peaks. Each amplitude spectrum contributes one of the *N*_v_ peaks for an ROI. Each ROI contributes one quantile value. We report the intensities of the second-strongest peak because the strongest peak in the (unnormalized) amplitude spectrum gives only the total number of atoms inside the ROI. We document in the supporting information that the real-space method yields signatures whose signal-to-noise ratio does not reduce substantially when removing atoms randomly.

For an increasingly strong displacement of the atoms, though, Fig. 3 ▸ shows a substantial reduction in the signal-to-noise ratio. For a standard deviation of σ_*z*_ = 0.25 Å along the data set main axis, the resulting displacements already exceed those of thermal lattice vibrations (Lonsdale, 1948 ▸). Nevertheless, the peaks remain strong against the background. Even for σ_*z*_ = 0.5 Å the intensity peaks remain detectable but at half the signal-to-noise ratio. In this case already half of the atoms are displaced by more than 20% of the lattice constant. For even stronger displacements the peaks eventually disappear, when approximately 20% of the atoms are displaced statistically by distances of more than half the unit cell. These results suggest that the signatures still show distinct intensity peaks, even for data sets with displacements which are stronger than thermal lattice vibrations. This offers potential for automatic indexing and orientation mapping.

We have implemented such analyses for arbitrary space groups. We can learn from the examples above that the key quantities to inspect when distinguishing different crystal structures in a noisy data set is how the position deviations (due to noise) compare to the distribution of the nearest- and higher-order nearest-neighbour spacings of the respective atom types in the unit cell.

### 3.2. Verifying the indexing for synthetic data and single crystals   

Consequently, we take the next step of the verification and attempt the indexing of a single crystal. Again synthetic data sets are used because these can be created in arbitrary orientation and thus enable a quantitative assessment of how precisely and accurately the orientations are identifiable. Specifically, four single-crystalline aluminium data sets were created. All four data sets represent a structure with approximately 20 × 10^6^ atoms in orientation (φ_1_ = 8.0°, Φ = 8.0°, φ_2_ = 8.0°). Different displacements of atoms (σ_*z*_ = 0.00, 0.25, 0.50 and 0.75 Å) were probed. A total of 1 × 10^4^ ROIs were placed randomly.

**Figure 4:**
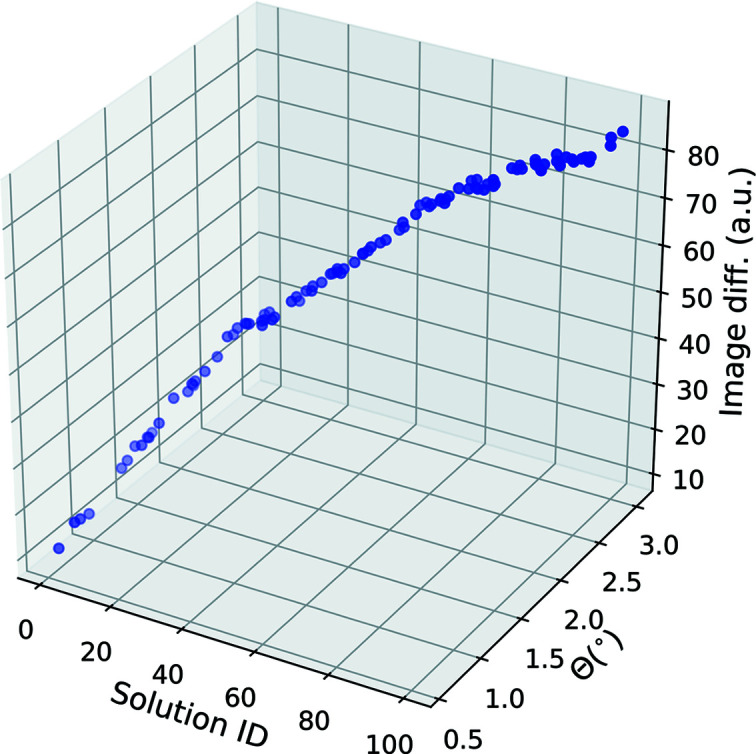
Verification of the indexing for a single ROI and the synthetic single crystal. The plot shows the image difference (image diff.) of the 100 best matched solutions as a function of the solution ID and the disorientation angle. ‘Best matched’ in this context means rotated versions of the reference that match with the signature of the ROI. The disorientation is computed between the orientation which the rotated version of the reference represents and the true orientation of the single crystal.

Fig. 4 ▸ documents the key results when indexing the signature of a single such ROI against a single candidate: an image difference value (image diff.) which quantifies how strongly the signature of the ROI differs from a particularly rotated reference (signature) of the candidate. Lower values indicate a better match of the image intensity peaks. Given that in this verification we prescribe the orientation of the single crystal, it is possible to compute the difference between the orientation represented by the particular rotated reference and the true orientation of the single crystal.

The results demonstrate that *PARAPROBE* outputs the true orientation precisely, accurately and consistently. In fact, the solution with the lowest identification has the lowest image difference and the lowest disorientation angle (Θ). Our method also shows that multiple similar well matched solutions exist, all of which register rotated references, representing the crystal structure candidate in different orientations. Some of these have a slightly higher disorientation angle (1–3°), suggesting that they are slightly rotated signatures of the true orientation or its symmetrically equivalent variants.

**Figure 5:**
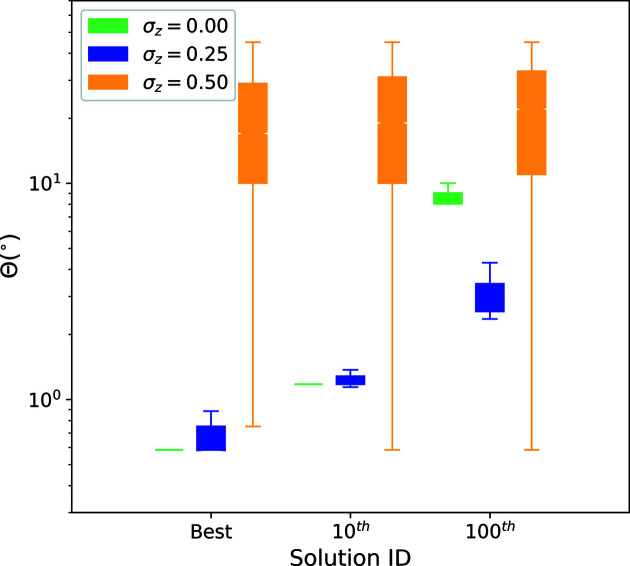
Verification of the indexing for all the ROIs of the synthetic single crystal. The best and two less well matched solutions are chosen for each ROI and characterized with respect to their disorientation to the true orientation of the single crystal. Stronger disorientation indicates poorer indexing. Stronger scatter indicates less robust indexing. The results confirm that it is possible to index robustly for up to σ_*z*_ = 0.25 Å, σ_*x*_ = σ_*y*_ = 2σ_*z*_ displacement (standard deviation).

Having discussed a single ROI, we next focus on the ROI ensemble and investigate if the true orientation is recoverable in all regions of the single crystal. Fig. 5 ▸ confirms that *PARAPROBE* recovers this information. The figure summarizes statistics for all ROIs. The results reveal how the particular well matched solutions scatter for a given amount of positional noise. Again, the disorientation angle between the known solution and the calculated solution in the ROIs quantifies the indexing quality. The disorientation should ideally be the same for each ROI and close to the resolution of the orientation grid 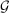 (∼1°) because of probing a single crystal. The results signify that the method indexes correctly because strongly disoriented best solutions are not found. Reassuringly, the poorer solutions have a consistently higher disorientation angle. We also observe that indexing is possible as long as the noise remains below σ_*z*_ = 0.5 Å (in this example).

### 3.3. Verifying the indexing of polycrystals   

As the last verification, we attempt to index a synthetic polycrystal. For this purpose we built a needle-shaped synthetic data set with approximately 200 × 10^6^ atoms (Kühbach *et al.*, 2019*b* ▸, 2021 ▸). Grains with an average spherical equivalent diameter of 200 Å were created by placing seed points of a three-dimensional Poisson–Voronoi tessellation (Okabe *et al.*, 2000 ▸) inside the data set. After assigning a random orientation for each grain, we filled each corresponding Voronoi cell with a local aluminium structure and a (random) orientation for this structure.

The practical advantage of this verification study is that the shape of the grains is rigorously defined. This enables us not only to compute the location of each boundary between any two cells (grains) but also to compute the locations of the junctions between the interfaces. This offers a unique opportunity to quantify how much of the volume of each ROI lies within a particular grain (Voronoi cell). We define this volume fraction as ζ_*k*_, *i.e.* how large a volume fraction of an ROI is occupied by a grain *k*. Values of ζ_*k*_ = 1.0 encode the fact that the ROI is completely embedded in grain *k*. A value of ζ_*k*_ = 0.5 means that only half of the ROI volume is covered by grain *k*. Three realizations of this data set were created, differing only in their positional noise (σ_*z*_ = 0.00, 0.25 and 0.50 Å) but using the same seeds for the grains. We scanned a 3D grid of ROIs with (10 Å)^3^ spacing.

**Figure 6:**
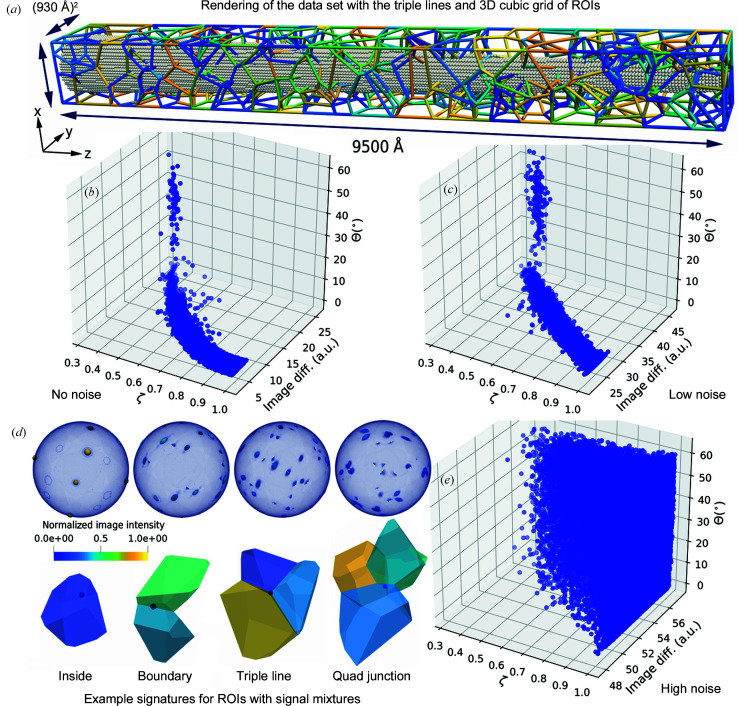
Final verification with a synthetic polycrystal. The ROIs are shown as grey spheres. The wire-frame diagram in panel (*a*) represents the triple lines between the grains (Voronoi cells) coloured by grain ID. Panels (*b*) (σ_*z*_ = 0.00 Å), (*c*) (σ_*z*_ = 0.25 Å) and (*e*) (σ_*z*_ = 0.50 Å) display for all ROIs of the data set the disorientation to the individual true orientation of each ROI. The blue points display the disorientation (angle) as a function of the image difference and the signal contribution (ζ) from the grain with the highest signal contribution. The results confirm that fully automated indexing is possible as long as the positional noise remains lower than σ_*z*_ = 0.5 Å. For ROIs at grain boundaries and junctions it is already evident from (*b*) that the solution quality deteriorates systematically the more strongly the neighbouring grains mix signal contributions into the signature. Panel (*d*) shows exemplar cases of the signatures and the 3D geometry of ROIs in different relative locations to the grain boundary network (inside, boundary, triple line and quad junction).

Fig. 6 ▸ depicts the interface network of the polycrystal (coloured wire frame) and the 3D ROI grid (grey spheres). We use the notation and quantities that were introduced with Fig. 4 ▸. Panels (*b*), (*c*) and (*e*) in Figs. 6 ▸ depict a collection of points. Each point represents the image difference (image diff.) for the closest matching orientation which *paraprobe-indexer* suggests for each ROI. Each point shows one ROI. For each ROI we computed the strongest volume contributions ζ (on the *x* axis). We decided in this verification study that the grain with the largest volume fraction defines the reference grain for the ROI. With this reference grain, it is possible to compute the disorientation angle between the orientation suggested by *paraprobe-indexer* and the true orientation. Figs. 6 ▸(*b*), 6 ▸(*c*) and 6 ▸(*e*) show the solution quality (image diff. on the *y* axis) as a function of the disorientation (angle) to the true orientation (on the *z* axis) and the strongest volume contribution ζ (on the *x* axis). Now, one can compare the indexing success for no positional noise [Fig. 6 ▸(*b*)] with the results for a low [Fig. 6 ▸(*c*)] and a high amount of positional noise [Fig. 6 ▸(*e*)]. Fig. 6 ▸ summarizes the key achievement of this work: it is possible to index APT data sets with fully automated methods, comparable to 3D orientation mapping for scanning electron microscopy (SEM) and EBSD, provided that the reconstruction is sufficiently accurate. Most ROIs are solved accurately and precisely, many with better than 1° angular resolution, thanks to the combination of the finite-element mesh and the orientation set 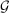.

This is an improvement compared with previous studies for several reasons. Not only is it the first work to use a fully automated protocol for rigorously quantifying the effect of signal mixture, but our approach even works for specimens with 200 × 10^6^ atoms and executes substantially faster because of sequential optimization combined with parallelization. This will be proven in the *Benchmarking* section[Sec sec3.6]. We do not rely on manual analyses (Liddicoat *et al.*, 2010 ▸), nor do we work exclusively in detector space (Yao, 2016 ▸; Wei *et al.*, 2018 ▸, 2019 ▸) or need to have an elemental segregation at the interfaces (Felfer *et al.*, 2015 ▸; Zhou *et al.*, 2021 ▸).

Having verified the functioning and consistency of the tool, the results in Fig. 6 ▸ show that the indexing of a data set fails systematically beyond a certain amount of positional noise (Vurpillot *et al.*, 2001 ▸; Breen *et al.*, 2015 ▸). There are two contributions which act concomitantly to cause indexing failures:

(i) The signal-to-noise ratio decreases with increasing positional noise. Thereby, all possible peaks with which a signature is indexed get weaker. This reduces the discriminatory power of the image comparison method, and thereby the capability of the algorithm to identify, and still reliably, as few as possible of the candidates which match the rotated references.

(ii) The results demonstrate that indexing fails first close to interface junctions, *i.e.* for ROIs where the signal comes from multiple crystals. Selected examples for grain boundaries, triple lines and higher-order junctions are shown in Fig. 6 ▸(*d*).

An explanation for incorrect indexing is evident in Fig. 6 ▸(*b*). We generated a nanocrystalline aggregate with a quasi-random texture. Therefore, most grain boundaries are of high-angle character (Mackenzie, 1958 ▸). In effect, only one orientation of a grain pair decodes a low disorientation, while the other disorientation is centred around the peak of the Mackenzie distribution.

The values for the standard deviations (σ_*xy*_ = 2σ_*z*_ with σ_*z*_ ≤ 0.75 Å) are comparable to those used by Vurpillot *et al.* (2001 ▸) and Breen *et al.* (2015 ▸).

### 3.4. Assessing the significance of signal mixture   

These examples quantify how strongly a certain amount of signal mixture reduces the indexing quality. For reconstructed data sets from real APT experiments, making such a rigorous comparison is very difficult without having access not only to correlative results in general but also to reconstructions of the interface network with ångström precision. For the above synthetic polycrystal, though, we can quantify from which grains each ROI obtains its crystallographic signal. For this purpose, we implemented an exact numerical computational geometry method which is detailed in the supporting information. This method uses a tetrahedralization of each Voronoi cell polyhedron with which we computed the accumulated intersection volume between each spherical ROI and the respective set of tetrahedra (Si, 2015 ▸; Strobl *et al.*, 2018 ▸). This enabled computation of the grain-specific intersection volume fraction ζ_*k*_ for each ROI.

The effect of signal mixture close to interfaces, here exemplified by grain boundaries, is best understood for the case of no noise [Fig. 6 ▸(*b*)]. There are only a few ROIs (blue dots) with ζ < 0.5, *i.e.* for which the signal comes from at least two grains. Only for these ROIs is the disorientation angle much higher than 1° (the resolution of the grid). The reason that the indexing fails here is because the signatures have too complex a mixture of intensity peaks, as shown for the example cases in Fig. 6 ▸(*d*). Therefore, indexing with a signature from a single crystal yields an arbitrary match in favour of one of the neighbouring grains, if any. The details are dependent on the exact intensity distribution and the individual signal contributions. In effect, the above verification highlights that to index reliably at interfaces needs further work towards *e.g.* advanced pattern matching or an iteratively refining indexing algorithm, a situation which is similar to that for electron diffraction methods (Wright *et al.*, 2014 ▸; Britton *et al.*, 2018 ▸).

We are aware that microstructures which get instantiated from Poisson–Voronoi tessellations have flat interfaces. To arrive at more realistic interface networks, it is possible to replace the structure synthesis in favour of more advanced protocols from the continuum microstructure modelling community, for instance via interfacing to tools such as *DREAM.3D* (Groeber & Jackson, 2014 ▸). This would add interface facets with different curvatures. Consequently, the distribution of ROI volume among the grains will change. This should pose no fundamentally new challenge, though, for the question of whether indexing is possible or not.

### 3.5. Application to experimental APT specimens   

#### 3.5.1. Aluminium bicrystal   

Finally, we applied the tools to two experimental APT data sets with strong crystallographic information. The first specimen was a technically pure single-phase aluminium bicrystal with a total of 48.7 × 10^6^ ions. The data set was characterized previously in substantial detail (Wei *et al.*, 2019 ▸). The data set was crystallographically calibrated, according to settings in the supporting information. Signatures were processed for several lattice plane families, {002}, {220} and {111}, for each ROI. The ROIs have a radius of 20 Å. ROIs were placed on a cubic ROI grid.

We implemented an iterative approach to refine this grid efficiently to pinpoint at which positions the crystallographic signal is particularly strong. Specifically, a local octree-like grid refinement was implemented. In the first iteration, the data set was scanned with a coarse cubic ROI grid with (20 Å)^3^ spacing. The distribution of signature intensity values for each signature and every ROI is analysed to identify those positions where the maximum intensity per signature exceeds a threshold value (here choosing κ ≥ 0.75 normalized image intensity). In a second iteration, we performed a local refinement of these ROIs by splitting the corresponding ROI grid cell into 5^3^ cells and placing that number of new ROIs at the respective centre of each ROI from the previous iteration.

We discuss the results for the {002} signatures here in the main paper, while the results for the {220} and {111} signatures are included in the supporting information. Synthetic data sets for aluminium single crystals from the verification were taken as the reference signatures. These were also computed specifically for {002}, *i.e.* composed from the peaks of the same bins.

**Figure 7:**
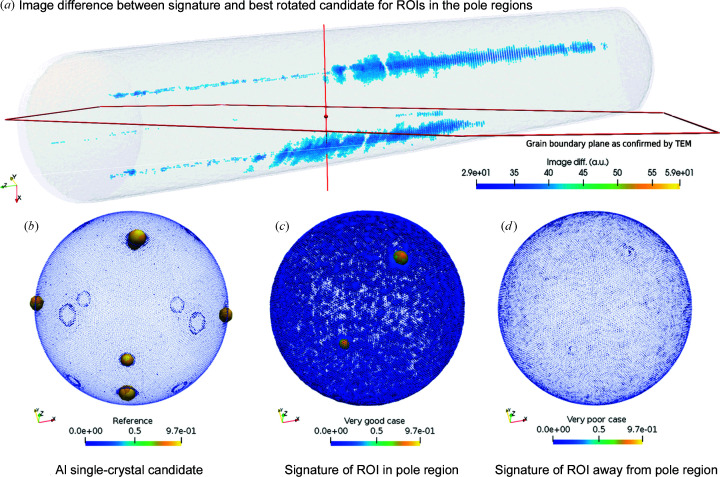
Quantification of the crystallographic signal quality within the experimental data set of the aluminium bicrystal (Wei *et al.*, 2019 ▸). (*a*) Our methods quantify that the crystallographic signal is strongest in the pole regions. (*b*), (*c*), (*d*) Comparison of the signature of (*b*) the reference (a synthetic aluminium single crystal) with observations for ROIs with (*c*) a particularly good (strong signature) or (*d*) a particularly poor (weak signature) signal quality. Thresholding via the image difference reveals that only for ROIs along the pole regions [turquoise–blue tube in panel (*a*)] are the signatures strong enough to pick up crystallographic information. Due to strong lateral distortions, not all of the expected peaks from the theoretical signature appear, even in the good signatures. Therefore, it is possible to output only information about the possible orientation fibres, not the specific orientation.

Fig. 7 ▸ shows that, for the data sets from real APT experiments on pure aluminium, our method is capable of extracting the crystallographic information content throughout the entire data set, volumetrically and in an automated manner. Fig. 7 ▸(*a*) displays a rendering of the reconstructed data set (grey shading) and the grain boundary (confirmed by correlative TEM; Wei *et al.*, 2019 ▸). Thresholding was used to visualize those regions in the data set where our method suggests that the crystallographic information content is highest – here for {002} in each of the adjacent grains. Given that these pole regions make up approximately only a tenth of the entire data-set volume, our adaptive grid refinement enables numerical costs to be cut where the signal is very likely to be low and invest these computations better in those regions where the signal quality is higher.

However, the spatial resolution is generally not sufficient to pick up multiple sets of planes within a single ROI, as Figs. 7 ▸(*b*), 7 ▸(*c*) and 7 ▸(*d*) illustrate. Fig. 7 ▸(*b*) shows the reference, the synthetic data set representing an aluminium single crystal, where numerous peaks corresponding to different sets of lattice planes are shown. Fig. 7 ▸(*c*) shows an example of a particularly good case from an ROI within the {002} pole region where the signature has two clear intensity peaks (yellow dots) opposite each other, confirming that two sets of planes are detectable within this ROI. However, compared with the signature of the reference in Fig. 7 ▸(*b*), the four other strong peaks (yellow) are missing in Fig. 7 ▸(*c*).

Fig. 7 ▸(*d*) also shows that, in most cases, the signatures have an insufficient signal-to-noise ratio. Virtually no peaks are detectable in these signatures measured outside the pole regions. Pitting this against the above verification of the real-space method, we can conclude either that the reconstruction quality in these regions is too low to recover the orientation of the crystal or that the respective lattice plane set cannot be detected due to geometric constraints during an APT measurement.

Such cases of missing peaks in the measured signatures makes unique indexing of the orientation based on the information in a single ROI impossible. One would have to analyse at least a second signature for a different set of planes {*hkl*} in another pole region to recover the orientation of the grain. While Fig. 7 ▸(*a*) clearly shows that the dominant {002} planes have been detected in each grain, other faint signals from other poles can also be observed. Combining this information would be sufficient to establish the crystallographic orientation of each grain relative to the detector.

#### 3.5.2. Al–Li–Mg–Ag alloy   

The second experimental example generalizes the above findings for data sets with second-phase precipitates and covers the situation when these precipitates are small enough to pose challenges with respect to finite counting effects. This is especially the case when the ROI and the precipitate radius are of the order of a few nanometres. The data set was reconstructed from measuring an Al–Li–Mg–Ag alloy specimen with a dispersion of Al_3_Li δ′ precipitates (Villars & Cenzual, 2016*a* ▸) (approximate radii 22–39 Å) inside a single-crystalline matrix. The specimen was reconstructed and characterized previously in detail (Gault *et al.*, 2012*a* ▸). This particular data set is a reconstruction from a specimen that was annealed for 8 h at 423 K. We scanned the data set with a (20 Å)^3^-spaced ROI grid (*R* = 20 Å). The signatures were compared against single-crystalline references for aluminium, lithium and Al_3_Li, using peaks specific for {002}, {220} and {111} lattice plane sets for the individual crystal structures and substructures. Again, we refined the ROI grid once. All results for the grid refinement are available in the supporting information.

**Figure 8:**
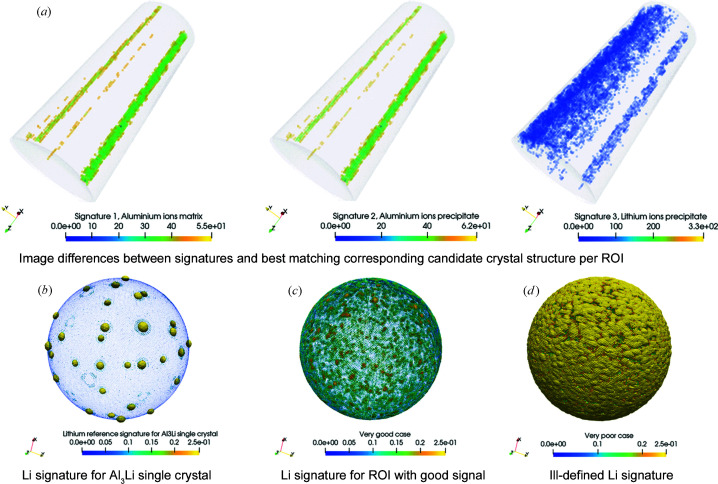
Quantification of the crystallographic signal quality within the experimental data set for the Al–Li–Mg–Ag alloy specimen (Gault *et al.*, 2012*a* ▸). (*a*) Thresholding via the image difference between the three individual signatures per ROI highlights again that the crystallographic signal is strongest in the pole regions. (*b*) The signature of the reference for the L1_2_ crystal structure, here quantified with the atoms of the lithium substructure. (*c*) This reference is compared with an example ROI with signatures that were among the strongest of all detected. (*d*) The last results are compared with an example ROI with ill-defined signatures because of limited lithium counts.

As an example, Fig. 8 ▸ summarizes the main findings for a discussion of the {002} signatures. The automatic approach detects regions in the data set which have a low image difference between the reference and the signature, indicating a stronger retained signal in the pole regions than elsewhere in the data set.

An example of finite counting effects is shown in Fig. 8 ▸(*d*). The example describes to which minimum atom count a crystallographic analysis with the real-space method can be pushed for a single ROI. The example signature shows strong intensity for virtually all projected directions. An inspection of the individual SDMs confirms that this is caused by spurious occupation of the SDM because the ROI contains few lithium atoms. Consequently, the FFT translates such a noisy and ultimately even skewed (Haley *et al.*, 2019 ▸) histogram into structure. The resulting intensity in the inspected bins of the amplitude spectra is either very low or very high. In the example presented here, the intensity is close to 1.0, *i.e.* almost as high as the DC components of the normalized amplitude spectra for many ROIs (see the diagram of the signal intensity versus atom count for the Al–Li–Mg–Ag data set in the supporting information). In effect, indexing becomes an ill-posed task.

Observation of such finite counting effects, in combination with spatial noise, pinpoints the key difference between algorithmic recovery of structural information from the reconstructed point cloud of a noisy APT data set versus applying structure identification algorithms to electron diffraction microscopy data: near-atomic resolution is not resolution on the scale of thermal lattice vibrations.

This concludes our analysis of methods for extracting signatures that capture the long-range periodic arrangement of atoms in 3D point cloud data based on the real-space method. With this we have resolved a remaining gap in the understanding of the real-space method (Araullo-Peters *et al.*, 2015 ▸). We have also delivered a so far missing fully automated method for indexing crystal structure and orientation from noisy point cloud data in experimental data sets. It was suggested recently (Haley *et al.*, 2019 ▸) that this is of interest for APT.

By developing a more performant set of tools and strategies for rigorously testing these, we have opened the door for a more productive high-throughput workflow within atom-probe crystallography. As a combination of open-source Python scripts and compiled scientific computing tools, our work can assist experimentalists in knowing which regions contain crystallographic signal and also quantify the relative signal strength of this information. This helps to supplement atom-probe crystallography studies which characterize poles in detector space or extract interfaces between crystals via elemental segregation. To conclude, such uncertainty quantification can help to improve open data exchange in the quest to enhance reconstruction algorithms.

### 3.6. Benchmarking   

#### 3.6.1. Real-space method   

**Figure 9:**
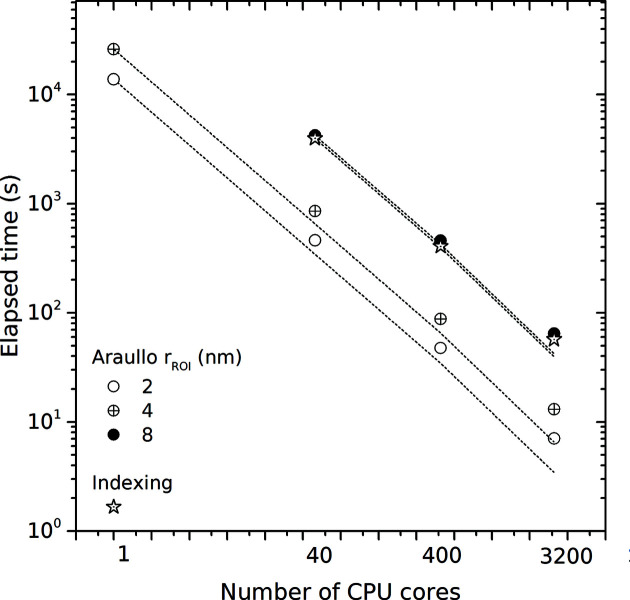
Elapsed time results for the same setups processed with an increasing number of CPU cores, demonstrating the strong scalability of the *paraprobe-araullo* tool. Acquisition with 80 Å (filled black circles) took approximately the same time as indexing (open star symbols). The straight dashed lines compare the results with the theoretical case of ideal linear scaling. These benchmarks always processed 1 × 10^4^ ROIs for the synthetic aluminium single crystals. Different ROI radii from 20 to 80 Å are compared for the same binning *m* = 10.

For practitioners it only remains to document the performance and scalability of the tools. To quantify the strong scalability (Kühbach *et al.*, 2021 ▸), we executed the above case studies with an increasing number of CPU cores or GPUs. The results are shown in Figs. 9 ▸ and 10 ▸, respectively. The dashed lines are linear extrapolations of the elapsed time reduction under the assumption that adding more CPU cores or GPUs results in a proportional reduction in the elapsed time (Amdahl, 1967 ▸).

If executed sequentially (one CPU core), the benchmark with the real-space method took 3 h and 50 min for *R* = 20 Å and solving 1 × 10^4^ ROIs. Using 40 CPU cores of a single node brings down the elapsed time to less than 8 min. This is a strong-scaling multi-threading efficiency of at most 76%. Using more cores results in higher productivity. As an example, solving the same benchmark above with 3200 CPU cores takes 13 s. This is an approximately 2000-fold performance increase or 63% strong-scaling efficiency. Two studies report performance data for the real-space method (Araullo-Peters *et al.*, 2015 ▸; Haley *et al.*, 2019 ▸), although they support only sequential execution. The results are difficult to compare with the present findings because different soft- and hardware and different settings were used. It seems, though, that these two studies are at least sequentially in the same order of performance. By contrast, our approach is scalable.

#### 3.6.2. Reciprocal-space method   

**Figure 10:**
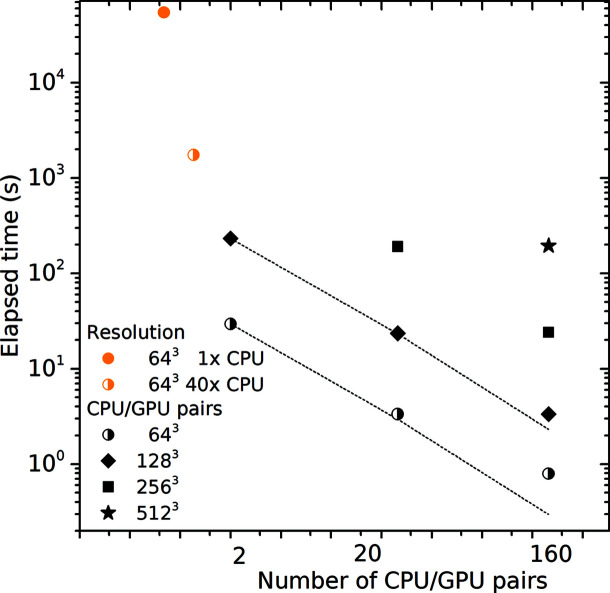
Elapsed time results for the same setups processed with an increasing number of CPU/GPU pairs, showing the strong scalability of the *paraprobe-fourier* tool. Different reciprocal-space resolutions (64^3^ to 512^3^) were tested. Straight dashed lines compare the results with linear scaling. The benchmarks probed 1 × 10^4^ ROIs with *R* = 20 Å and the synthetic aluminium single crystals.

The reciprocal-space method was executed with CPU multi-threading and alternatively with GPU processing (Fig. 10 ▸). For a set of 1 × 10^4^ ROIs with a radius of *R* = 20 Å and resolving the reciprocal space with an 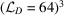 grid, the computation takes 15 h and 4 min when using a single CPU core. With 40 cores, the results are ready after 29 min, thereby documenting a strong-scaling efficiency of 78%. Using a single GPU, though, outperforms the 40 CPU cores by easily an order of magnitude. Using 160 GPUs of the TALOS cluster enabled us to complete the benchmark in 0.8 s for a reciprocal-space grid with 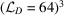 points, and in 200 s for 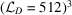 points. Tapping such, so far unused, resources enables a hitherto inaccessible uncertainty quantification for APT data.

## 4. Discussion   

This work is not complete without a discussion of the effects of contained crystal defects on the computed signatures and bridging between our and existing work on indexing crystal structure and orientation developed by other (microscopy) communities. Building these bridges is the purpose of this final section to envision possible strategies either to inspire development in these fields or to improve the above methods in the future.

### 4.1. Addressing preferential field evaporation, crystal defects, pseudosymmetry and solutes   

The algorithms presented here have not directly addressed the influence of preferential field evaporation effects and crystal defects which influence the local reconstruction of a repeated structure, and this remains outside the scope of the present article. The (indexing) performance of the algorithm is fundamentally linked to how closely a crystal structure is locally reconstructed, and it follows that the above influences will generally cause a loss in local recoverable crystallographic information content (Jenkins *et al.*, 2020 ▸; De Geuser & Gault, 2020 ▸; Gault *et al.*, 2021 ▸). A notable exception is the evaporation behaviour around low-index facets on the specimen which show as low-density poles on the detector. In these regions, lateral resolution is degraded by local magnification effects, but the highly ordered evaporation sequence in these regions works to improve the depth resolution and the detectable crystallographic signal (Gault *et al.*, 2008 ▸). The other degrading influences can be mitigated to some extent by appropriate ROI volume selection – the larger the volume, the lower the analytical resolution but the easier it is to detect average structure periodicity with SDMs.

In general, structure defects such as dislocations can be largely mitigated, but their influence becomes more significant around lower-angle grain boundaries with a high density of geometrically necessary dislocations and an increased relative volume fraction of an ROI probing defects. Local magnification around interfaces will cause degradation up to approximately 2 nm from these features, but the crystal structure and orientation of neighbouring grains can usually still be quantified if they contain at least two plane families (usually at corresponding poles) and are greater than several cubic nanometres.

Correct crystallographic indexing can also be challenging in cases of low-symmetry crystals and for other conditions where two signatures are difficult to distinguish in practice at a given resolution. Such degenerate cases are possible, for instance, when the features in the signatures are very similar, or when inspecting pairs of signatures from differently oriented crystals of the same or different crystal structure candidates. Not only the unit-cell dimensions for the same space group but also different space groups or crystal orientations can in general lead to combinations which will probably need more refined approaches than those we have discussed above. In the SEM/EBSD community these challenges are related for example to resolving pseudosymmetry. This is a known limitation for all crystallographic characterization techniques (Pang *et al.*, 2020 ▸), and is even more challenging in the case of APT crystallography where more limited crystallographic features are typically resolved and higher levels of positional noise and missing ions are faced. Care must be taken in the interpretation of the results and in the confidence of the output indexing, which will become worse with lower-symmetry crystal structures and where fewer lattice plane families are captured for a given grain.

Note that a partial replacement of atoms of the host structure with substitutional and/or interstitial atoms is conceptually no different from thinning out a structure of host atoms. Thus, signatures for a given crystal structure with no missing atoms but solutes instead are expected to be similar to the signature of the same structure with missing atoms. Therefore, the presence of solutes ultimately has an additional detrimental quantitative effect on the indexing quality if an ROI is already locally depleted of atoms because of a high fraction of missing ions within the reconstructed data set.

### 4.2. Spherical harmonics methods to represent the signatures and index them   

Indexing signatures against a library of precomputed rotated references is a brute force approach because it demands for each ROI the processing of a large set of rotations and a large number of image intensities. Instead, it might be more efficient to use alternative methods like spherical harmonics to solve this image registration task (Makadia *et al.*, 2006 ▸; Makadia & Daniilidis, 2006 ▸) and blend this with methods that were proposed recently in the (X-ray diffraction) texture and SEM/EBSD community (Hielscher *et al.*, 2019 ▸; Lenthe *et al.*, 2019 ▸).

The key idea reads as follows. An image, carrying the crystallographic information, is compressed into a reduced-order description. Next, it is projected into a mathematical space in such a way that particular mathematical rules can be applied to solve both the registration and the orientation task more efficiently. Assessing such a method and comparing it with ours is worth its own careful analysis. Therefore, we explore in this paper only whether it is possible to create such a reduced-order description of the signatures.

As a key requirement, a series expansion may be used to fit the intensities of the signature. The weights, or coefficients, of the series expansion afford a reduced-order description of the spherical image. The values of the series expansion are intended to vary sufficiently in order to fit to a generalized distribution across the surface of a sphere. This is an application for discrete spherical harmonics.

**Figure 11:**
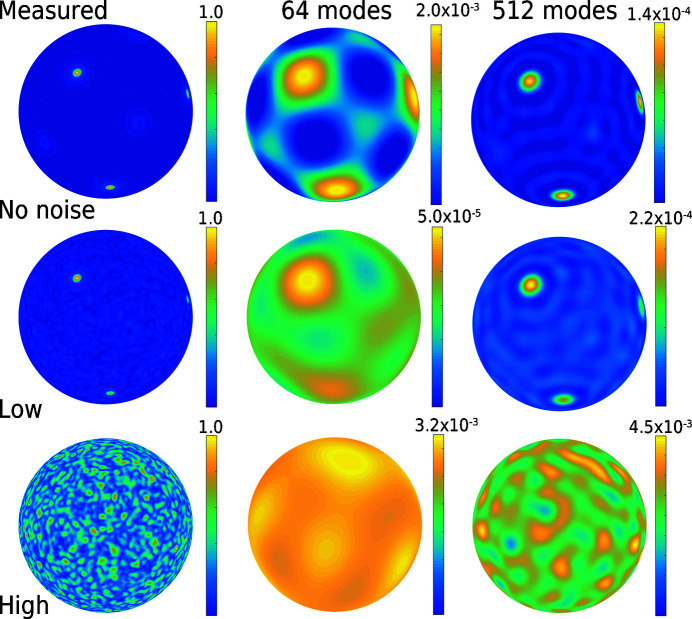
Preliminary results on approximating the signatures for ROIs probed in the synthetic aluminium single crystal for different strengths of positional noise. The colour bars display the image intensity using individually linear scales with values that range from 0.0 to the shown individual maximum intensities. Spherical harmonics approximated spherical images (of the signatures in the left-hand column). We compared the approximations for 64 and 512 spherical harmonics modes.

Here, we employ a finite-element approach and evaluate sequential MATLAB code to find the corresponding weights of the discrete harmonic series expansion. Specifically, we adapt a strategy to fit spherical harmonics to lattice-strain pole figure intensities (Wielewski *et al.*, 2017 ▸). The key mathematics are recapped in the supporting information because we only replace the pole figure intensities with image intensities of the signature. As an example, we inspected spherical harmonics descriptions of several signatures from the synthetic aluminium single crystals in (φ_1_ = 8.0°, Φ = 8.0°, φ_2_ = 8.0°) orientation, using the single crystal reported above for verification of the method. The results are summarized in Fig. 11 ▸.

First, we focus on the image matrix in Fig. 11 ▸ to check which information content of the signatures the spherical harmonics recover. The rows compare the signatures (left-hand column) with the fitted signatures (right-hand column). We compare, for increasingly strong positional noise, a perfect crystal (σ_*z*_ = 0.00 Å, left-hand column, top row), a crystal with low noise (σ_*z*_ = 0.25 Å, left-hand column, middle row) and a crystal with high noise (σ_*z*_ = 0.75 Å, left-hand column, bottom row). The columns of the matrix compare these signatures with the approximation of the intensities via spherical harmonics using 64 or 512 harmonic modes, respectively. The spherical harmonics pick up the location of the intensity peaks already with a low number of modes. However, this comes at the cost of substantially smeared out intensities and moderate approximation quality improvement for an increasing number of modes.

In turn, our explored combination of a fine FE mesh and orientation grid resolution resulted in angular accuracy and precision very close to the best so far achieved of 0.5°, as was shown experimentally in the correlative TKD microscopy and APT studies of Breen *et al.* (2017 ▸). Consequently, we wish that any proposed spherical harmonics algorithm should ideally be equally accurate and precise. In this regard, the results in Fig. 11 ▸ are preliminary. Nevertheless, they suggest that spherical harmonics could be a useful alternative for compressing signatures.

### 4.3. Methods from TEM as an alternative to index APT data   

We observe that the crystallographic images from the reciprocal-space method are almost equal in format to automated diffraction tomography (ADT) data sets from TEM diffraction studies. Algorithms have been developed in the TEM community (Campbell, 1998 ▸; Kolb *et al.*, 2007 ▸, 2008 ▸, 2011 ▸; Maia *et al.*, 2011 ▸) which output the crystallographic orientation for a known crystal structure. Ultimately, these algorithms are even capable of detecting the most likely crystal structure candidate. Observing the recent progress in the field of electron nanodiffraction (Zuo, 2019 ▸), this could be an option to explore in another interdisciplinary atom-probe crystallography study in the future. It is possible to post-process the signatures from the reciprocal-space method, resulting in compressed sparsified signatures. An example is a list of locations of high reciprocal-space signature intensity values. Such a description is what recent tools for precession electron diffraction methods [see Midgley & Eggeman (2015 ▸) for an overview of these methods] use for structure analysis. This observation makes another link of potential research between microscopy communities.

### 4.4. Artificial intelligence methods as an alternative to index APT data   

Similarities exist between the reciprocal-space method and recently proposed deep-learning approaches for identifying crystal structures (Ziletti *et al.*, 2018 ▸; Leitherer *et al.*, 2021 ▸). For example, both methods encode the structural information via an image of (direct) Fourier-transformed atom positions and formulate indexing as an image processing task.

Neither of the above methods nor the artificial intelligence methods work without computing a signature. In the artificial intelligence approach, these are the raw data and respective inputs for training and inference. It can be seen as a benefit of deep-learning approaches that the features of the signatures do not need to be encoded manually because they are evaluated as part of the feature mapping during training and inference. By contrast, in our indexing approach with the real-space method, it is necessary to define *a priori* from which peak(s) in the amplitude spectra the signature is composed. We have shown that this is possible for some crystal structures, provided there are no ambiguities. Resolving these can be more complicated, though, for arbitrary crystal structures, especially for those with a low symmetry.

A clear disadvantage of the above deep-learning approach is that it does not so far account for the relative orientation of the crystal. This holds at least as long as a descriptor like the smooth overlap of atomic potentials (Bartók *et al.*, 2013 ▸; De *et al.*, 2016 ▸) is employed, which is typically formulated as rotation invariant. At least technically such a limitation could be lifted, though, and combined with training on explicit sets of candidate orientations for each crystal structure via data augmentation.

Alternatively, deep learning could be combined with the above-mentioned spherical harmonics description. Blending our methods with artificial intelligence could help to improve the results in cases where the discussed signal mixtures at interface junctions are challenging. Nevertheless, it remains to be shown that the robustness of the above deep-learning methods also holds for noise levels as high as those inspected in this work.

## 5. Summary   

We have developed a method for extracting crystal structure and orientation volumetrically within reconstructed atom-probe tomography data sets. The tool works accurately and precisely. It is fully automated and scales strongly on parallel computers. The methods are delivered as open-source software to contribute to faster and more reliable atom-probe crystallography. Verification and validation studies with synthetic data sets and experimental APT specimens brought the following conclusions:

(i) Suitably modified, the method of Araullo-Peters and co-workers yields reproducible and robust crystallographic information for spatially uncorrelated missing atoms, or data sets where the atoms are displaced by as much as 10% of the lattice spacing out of their equilibrium positions.

(ii) Using a different data post-processing strategy, the method becomes a tool for identifying crystal structure and orientation. We verified and validated its functionality for noisy data sets from single crystals and polycrystals with an arbitrary grain-boundary network.

(iii) Software parallelization now enables analyses on computer clusters, using either CPUs alone or a combination of CPUs and GPUs. Benchmarks with at most 3200 CPU cores or 160 GPUs, respectively, delivered three orders of magnitude faster processing compared with previously reported sequential tools.

(iv) These achievements enable fast and reliable measurements of the local crystal structure throughout APT reconstructions where sufficient spatial resolution is present (usually the pole regions). This information can be used to calculate user-defined reconstruction parameters and subtle changes throughout an experiment to facilitate dynamic reconstruction protocols.

(v) A quantitative assessment of material points in close proximity to grain boundaries and triple lines addressed the deterioration of the crystallographic information due to signal contributions from multiple grains. Strategies for improvements were sketched and discussed in relation to alternative methods for crystal structure identification from the electron and X-ray diffraction communities.

(vi) The automated method was tested on an experimental aluminium bicrystal and an Al–Li–Mg–Ag alloy. Crystallographic signal was detected in the pole regions. In some cases, two sets of planes could be detected in a single ROI, but in most cases only one set of planes could be detected in each pole. In regions outside the poles, the method did not detect resolvable structure. Nevertheless, the method provides a fast and powerful way to detect and quantify latent crystallographic information within experimental APT reconstructions in three dimensions that until now has had practical limitations due to the high computational complexity, which we mitigate with highly performant parallelized and CPU- and GPU-optimized code.

## 6. Work distribution   

M. Kühbach designed the study, implemented *PARAPROBE* and led the writing of the manuscript. M. Kasemer contributed the geodesic finite-element mesh and MATLAB scripts for fitting signal intensities using spherical harmonics. A. Breen contributed the in-depth crystallographic analyses of the experimental results. All authors discussed the results and contributed to writing of the manuscript.

## 7. Related literature   

For further literature related to the supporting information, see Ayachit (2015 ▸), Bachmann *et al.* (2010*a* ▸), Bonnet (1980 ▸), Da *et al.* (2018 ▸), Frigo & Johnson (2005 ▸), Grimmer (1974 ▸), Gropp *et al.* (1998 ▸, 1999*a* ▸, 1999*b* ▸), Heinz & Neumann (1991 ▸), Intel (2019 ▸), Jeffers & Reinders (2015 ▸), Kaiser & Schafer (1980 ▸), Morawiec (2004 ▸), Portland Group (2020 ▸), Prabhu (2013 ▸), Reinders & Jeffers (2014 ▸), Reinhard *et al.* (2019 ▸), Schäling (2014 ▸), The CGAL Project (2018 ▸) and Ulfig *et al.* (2017 ▸).

## Supplementary Material

Additional details for the algorithm. DOI: 10.1107/S1600576721008578/yr5067sup1.pdf

## Appendix A Data and source code availability

The source code of the tools, hands-on tutorials in the form of Python/Jupyter notebooks for practitioners, and the configuration files and data sets for software developers are available as open-source supplementary material (https://gitlab.mpcdf.mpg.de/mpie-aptfim-toolbox/paraprobe.git; https://paraprobe-toolbox.readthedocs.io).

During the revision of the manuscript we addressed an inconvenience of the software design which is to have in total three tools for signature collection, one for RSP, one for FSP and one for indexing. As a part of the revision of the manuscript we opted to integrate these tools into one (http://gitlab.com/paraprobe/paraprobe-toolbox.git), which makes setting up workflows easier, replaces OpenACC with CUDA and supports importing crystal structures for tools like the *Atomic Simulation Enviroment* (Larsen *et al.*, 2017 ▸). Only this newer tool will be maintained in the future.
