# Supplementary material for: Open and strong-scaling tools for atom-probe crystallography: high-throughput methods for indexing crystal structure and orientation
Source: J Appl Crystallogr. 2021 Oct 1;54(Pt 5):1490–508. doi: 10.1107/S1600576721008578 (PMC8493626; doi:10.1107/S1600576721008578)
Supplement: Supplementary file 1 [file j-54-01490-sup1.pdf]

JOURNAL OF
APPLIED
CRYSTALLOGRAPHY

Volume 54 (2021)

Supporting information for article:

Open and strong-scaling tools for atom-probe crystallography: high-throughput methods for indexing crystal structure and orientation

Markus Kühbach, Matthew Kasemer, Baptiste Gault and Andrew Breen

On Open and Strong-Scaling Tools for Atom Probe Crystallography: High-Throughput Methods for Indexing Crystal Structure and Orientation — Supplementary Material —

Markus Kühbach,[a,b,c]* Matthew Kasemer,[d] Baptiste Gault[a,e] and Andrew Breen[f]

[a] *Max-Planck-Institut für Eisenforschung GmbH, Max-Planck-Straße 1, D-40237 Düsseldorf, Germany,* [b] *Fritz Haber Institute of the Max Planck Society, Faradayweg 4-6, D-14195 Berlin, Germany,* [c] *now at Humboldt-Universität zu Berlin, Structure Research & Electron Microscopy group, Department of Physics, Consortium FAIRmat, Newtonstraße 15, D-12489, Berlin, Germany,* [d] *Department of Mechanical Engineering, The University of Alabama, Tuscaloosa, AL 35487 United States,* [e] *Department of Materials, Imperial College London, Royal School of Mines, London UK, and* [f] *The University of Sydney, Australian Centre for Microscopy & Microanalysis, NSW 2006 Sydney Australia. E-mail: kuehbacm@hu-berlin.de*

1. Computational methods

1.1. Setting up atom probe crystallography workflows with PARAPROBE

Input and prerequisities In this section we will describe the workflow that we used with the initial version of the tools. All results for the main paper were computed using this version. The workflow has been simplified in the updated version of the tool. Users should consult associated Jupyter notebook examples on how these new tools can be used for programmatic crystal structure characterization using PARAPROBE. Setting up a workflow to the analyses in the main paper starts with defining four ingredients: a dataset (synthetically created or a reconstructed dataset from an APT experiment), ranging definitions, i.e. rules how to map mass-to-charge-state ratios to atom types, an ensemble of regions-of-interest ROIs, and a set of crystal structure candidates with individual definitions along which crystallographic directions one wants to probe, and hence which region(s) of the amplitude spectra to investigate. We worked with synthetic datasets as well as datasets from real APT experiments. Synthetic datasets were created using the paraprobe-synthetic tool (Kühbach *et al.*, 2021). The synthetic datasets were on the one hand used to build the datasets for verifying the tools and on the other hand used to compute the reference signatures for single crystals with specific crystal structure and orientation.

For processing experiments we imported reconstructed atom positions by transcoding either POS or EPOS files, respectively from The Integrated Visualization and Analysis Suite (IVAS) (Ulfig *et al.*, 2017; Reinhard *et al.*, 2019) into a Hierarchical Data Format (HDF5) file representation as it is required for PARAPROBE. For this task we worked with the paraprobe-transcoder tool. For ranging, PARAPROBE accepts ranging data and definitions that have been made with external tools from the APT community. Such ranging data define how to map from mass-to-charge-state ratios to (molecular) ion types. For ions that are composed of isotopes of a single

element (the case in this work) we treat ion types to be equivalent as atom types. For molecular ions a so-called atomic decomposition can be applied to map an ion type to a specific atom type provided an isotope of the requested target element is a component of the molecular ion. Specifically, we worked with the import functionalities of the paraprobe-ranger tool and read RRNG files, which is a common human-readable text file format used by atom probe microscopists to communicate ranging results. The ranging was created by the respective APT experimentalists within IVAS. The definition of the ROI ensemble and the crystal structure candidates is detailed in the main paper for each respective case study.

Workflow Quantifying crystallographic signal and indexing it via PARAPROBE is realised with a sequential workflow of PARAPROBE tools. Individually, these tools use parallelisation. The steps for the real space method were as follows:

1. We synthesise synthetic datasets for the crystal structure candidates, range these, and characterise their atomic architecture. Thereafter, we evaluate the amplitude spectra for each spatial distribution map (SDM) and decide which lattice plane stack to analyze. Based on this we decide which region of the amplitude spectra we need to probe and compose into signatures:
    (a) paraprobe-synthetic,
    (b) paraprobe-ranger,
    (c) paraprobe-surfacer,
    (d) paraprobe-araullo.
2. The analysis for the dataset to be indexed uses the above-created reference signatures for the crystal structure candidates:

(a) paraprobe-transcoder or paraprobe-synthetic, respectively depending on whether data come from experiment or are synthetic,

(b) paraprobe-ranger to accept the external ranging for the dataset,

(c) paraprobe-surfacer to compute the distance of the ROI to the dataset edge,

(d) paraprobe-spatstat to optionally characterise spatial statistics,

(e) paraprobe-araullo to compute the signatures for all ROIs,

(f) paraprobe-indexer to index the signatures against the rotated references for the crystal structure candidates.

Figure 1 summarises the resulting workflow and how the real space and reciprocal space methods, respectively are integrated into this workflow.

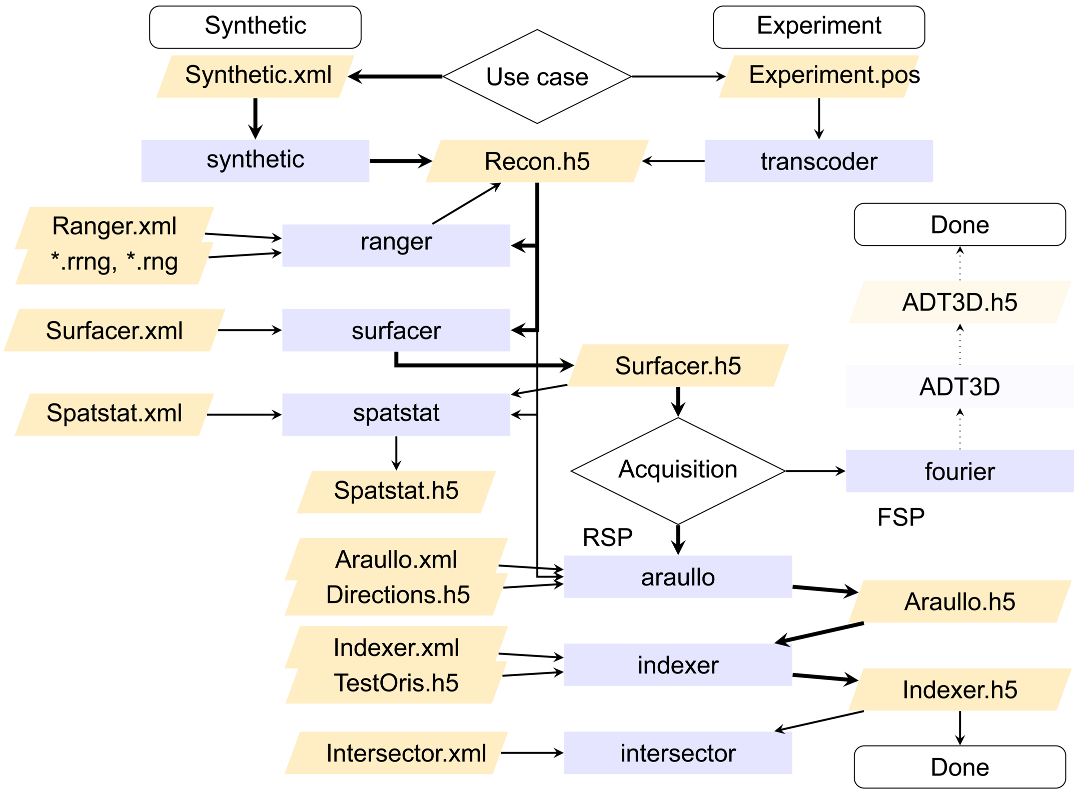

Fig. 1. Proposal for an automated workflow for indexing crystal structure and backing out crystal orientations locally. Thicker arrows follow the main steps of the workflow from the creation of a synthetic or the importing of a reconstructed dataset from an experiment to the acquisition of the signatures. RSP abbreviates the real space method. FSP abbreviates the reciprocal space method. Instead of a monolithic software, we connect multiple specialised tools (blue boxes) into a workflow. The analysis steps are controlled through XML configuration files. All heavy data are carried through the processing with HDF5 files.

The GitLab repository of PARAPROBE (Kühbach, 2020) comes with a set of Jupyter notebook tutorials which exemplified further details of this workflow. Users should inspect the latest version of PARAPROBE and the corresponding tutorial as the proof-of-concept implementation has been modified during the revision of the main paper.

1.2. Implementation details

Assuring that ROIs are completely in the dataset To assure this, we first binned each dataset into 10 Å cubic voxels. Then, we identified the interior voxels using methods previously described (Kühbach *et al.*, 2021). Regions-of-interest (ROIs) were placed only in such voxels. In addition, dense α-shapes of the point cloud were computed (Kühbach *et al.*, 2021). The resulting triangle hulls were evaluated for intersection with the ROIs. Only ROIs without intersections with the α-shapes were used. In combination, this ensures that the ROIs are fully embedded in the dataset.

Why geodesic spheres instead of a regular gridding of elevation/azimuth? Multiple strategies exists to define along which projection directions one probes for the real space method. Compared to a regular gridding of the elevation-azimuth space, as it was proposed in (Araullo-Peters *et al.*, 2012), we work with a geodesic sphere finite element mesh because it distributes the nodes more homogeneously over the surface of the unit sphere (Popko, 2012).

Controlled windowing of the spatial distribution maps (SDMs) We used a different binning strategy than the original authors (Araullo-Peters *et al.*, 2012) for preparing the SDMs before computing their fast Fourier transformations. Specifically, we pad the histogram on either side by one bin with zero counts to enable for any implementation of controlled signal windowing (Prabhu, 2013). In this work, we used a rectangular windowing function. Alternatively, Kaiser windowing could be used (Kaiser & Schafer, 1980) for instance.

Creation of test orientations for rotating the reference signatures We worked with a quasi-equidistant grid of test orientations in orientation space $\mathcal{G}$ with members $g \in \mathcal{G} \in SO3$ using the MTex texture toolbox (v5.0.3) for Matlab (Bachmann

et al., 2010*b*; Bachmann *et al.*, 2010*a*). Crystal symmetries were accounted for ($\frac{4}{m}\bar{3}\frac{2}{m}$). The angular resolution of the grid was 1°, resulting in a total of $N_g = 618324$ orientations. The rotations are evaluated in a pre-processing stage to build a library of rotated references (for each crystal structure candidate). Orientation differences were quantified using classical disorientation-based algorithms (Grimmer, 1974; Heinz & Neumann, 1991). Also these were implemented for cubic crystal symmetry only. Generalizations for lower symmetry crystal structures have been reported (Bonnet, 1980; Heinz & Neumann, 1991; Morawiec, 2004). The disorientation quaternion in this work is defined as a particular misorientation quaternion in the fundamental zone with the smallest rotation angle.

Indexing algorithm Running the real space method, which is referred to as RSP in the main paper, for an ensemble of ROIs yields a collection of spherical images, i.e. the signatures $\mathcal{S}_c^{msr}$ for each c-th crystal structure candidate. During indexing we evaluate nodal intensities using the paraprobe-indexer tool (Fig. 1). Indexing has three pre-processing and one indexing step. First, the intensities of the $\mathcal{S}_c^{msr}$ signatures and the rotated references $\mathcal{S}_c^g$ (signatures for the crystal structure candidates rotated by g) are normalised individually. The index g denotes the test orientation. Second, a user-defined sub-set of the FE nodes is defined (here $\hat{N}_v = 1000$) for each reference. We pick those nodes for which the signal is strongest. Third, orientation set $\mathcal{G}$ is used to rotate each of the $\hat{N}_v$ FE nodes and compute its closest neighbouring node $p_{v,c}^g$. The index v denotes a particular of the strongest intensities in decreasing order.

The result of the pre-processing steps is a look-up table. This look-up table encodes implicitly which nodal values of the signatures for the ROIs, i.e. $\mathcal{S}_c^{msr}$, have to be inspected to compare them with a specifically oriented version of a rotated reference. In effect, indexing reduces to a querying and comparing of image intensity values at pre-computed nodes. The difference Δ_c^g is quantified as the sum of squared image

intensity differences (Eq. 1). For each rotated reference in orientation g and each crystal structure candidate c we get one value for each ROI.

$$\Delta_c^g = \sum_{v=1}^{\hat{N}_v} (\mathcal{S}_c^{msr}|_{p_{v,c}^g} - \mathcal{S}_c^g|_v)^2 \tag{1}$$

Other descriptors could be used to quantify the differences between signatures. One of which is computing the cross-correlation between the signatures. Using this descriptor would result in higher numerical costs, though, because eventually all intensity values have to be compared.

Analytical intersection volume We state in the main paper to have developed a numerical exact method for computing the intersection between a spherical ROI and an arbitrarily shaped and oriented polyhedron. In this work, the polyhedra are the Poisson-Voronoi cells from the polycrystal construction that we interpret as the grains of the polycrystal. To the best of our knowledge there is no analytical formula to compute such intersection volume for arbitrary spatial arrangement of the geometric primitives.

However, there is a numerical method for computing the exact intersection volume of a sphere cutting an arbitrary tetrahedron (Strobl *et al.*, 2018) to within floating point precision. Recall, that one can decompose a convex polyhedron into a set of tetrahedra. In our case, we can thus decompose the volume of each Voronoi cell completely into a set of tetrahedra (constrainted tetrahedralization). These tetrahedra are boundary-conformant with the piece-wise linear complex that is represented by the faces of the Voronoi cell (Si, 2015). In effect, this enables a computation of the intersection volume between the ROI and each tetrahedron. This allows us to accumulate the individual values for ROI-sphere-tetrahedra-intersections to obtain a total intersection volume between a spherical ROI and each Voronoi cell of polyhedral grain k.

Sequential implementation tricks The numerical costs for computing fast Fourier transforms of the real space method are reducible with numerical libraries such as the Intel Math Kernel Library (Intel, 2019) or The Fastest Fourier Transform in the West (FFTW) (Frigo & Johnson, 2005) for the CPU, and cuFFT (Nvidia Corporation, 2019) for the GPU, respectively. Conjugate-even symmetry was assumed to reduce further the costs of the Fourier transformations. Single precision was used where possible. All data were packed into contiguous pieces of memory to reduce memory traffic and cache misses. Although we have not explicitly implemented it, one could also exploit that all histograms (SDMs) for a ROI have the same number of bins. In principle, this enables a batching of all the Fourier transformations for a ROI. Thereby, one can further reduce the numerical costs for loading intermediate values. We queried atoms for each ROI via multi-threading using Open Multi-Processing (OpenMP) commands.

1.3. Analysis of asymptotic computational costs

Real space method The numerical costs of the real space method are defined by four quantities: the number of ROIs N_{roi} in the ensemble, their radius R, the number of SDMs per ROI N_v, and the number of bins per SDM $N_b = 2^m$. For a dataset of volume V with homogeneous density of a single atom type ρ, and f being a geometrical constant, the average number of atoms in a ROI is $N_w = fR^3\rho$. Therefore, the asymptotic computational time complexity of collecting crystallographic signal with the real space method is:

$$\mathcal{O}_{Proj} = N_{roi} \cdot [fR^3\rho \cdot log(V\rho) + N_v[N_w + 0.5N_b log(0.5N_b) + 0.5N_b]]. \quad (2)$$

The summation terms account for the querying, the projecting, the Fourier transforming, and the peak identifying from the amplitude spectrum. Here we assume an algorithm with tree-based neighbour querying (Kühbach *et al.*, 2021) and amplitude spectra analysed within a selected frequency interval. Despite the numerical complex-

ity, there is substantial potential for parallelising those computations. In fact multiple computations are independent: each ROI, each direction, and the peak search for each amplitude spectrum. Our implementation is the first which taps this potential.

Reciprocal space method For the reciprocal space method we probed cubic subregions in reciprocal space at reciprocal space positions $\boldsymbol{k}$. Different resolutions were probed ($\mathcal{L}_D = 64$ to 512 for $\boldsymbol{k} \in [-2\pi, +2\pi]^3$). The asymptotic computational time complexity of the reciprocal method is:

$$\mathcal{O}(N_{roi} \cdot N_w \cdot {\mathcal{L}_D}^3), \tag{3}$$

where N_w is the number of atoms in the ROI. Given the resources available in 2001 (Vurpillot *et al.*, 2001), this rendered routine application of the method impractical. However, the computations are independent for every ROI and have low summation costs for every atom w and every reciprocal space position k. In combination with the CPU and especially GPU hardware improvements in the last 20 years (Hennessy & Patterson, 2012; Rauber & Rünger, 2013; Reinders & Jeffers, 2014; Jeffers & Reinders, 2015), this makes the proposal and method of Vurpillot *et al.* potentially attractive again for quantifying crystallographic signal.

Indexing Our implemented indexing method has a computational time complexity with two key contributions: a constant look-up table creation cost $\mathcal{O}_{lu}$ and indexing costs $\mathcal{O}_{idx}$. The creation costs are $\mathcal{O}_{lu} = N_c \cdot N_g \cdot \hat{N}_v$, in which $\hat{N}_v$ is the number of FE nodes at which image intensities are evaluated and N_c the number of crystal structure candidates. The indexing costs are $\mathcal{O}_{idx} = N_{roi} \cdot N_c \cdot N_g \cdot \hat{N}_v$. Both contributions offer substantial potential for parallel execution. The computation of a look-up entry is an independent task per entry. Equally, each ROI is independent and at this level also each orientation and phase candidate.

1.4. Soft- and hardware details

These methods were implemented as additional tools (paraprobe-araullo, paraprobe-fourier, paraprobe-indexer, paraprobe-intersector) into the open-source PARAPROBE toolbox. The tools were compiled with the Intel (v18.0.5.20180823) compiler using Skylake CPU-specific optimisation. Algorithms from the Computational Geometry Algorithms Library CGAL (The CGAL Project, 2018; Da *et al.*, 2018; Kühbach *et al.*, 2021) (v4.11.3) and Boost C++ (Schäling, 2014) (v1.66) were employed. When using the GPUs, code was compiled with the PGI (v19.10-0 LLVM) compiler (Portland Group Inc., 2020) using Skylake CPU-specific and GPU-specific optimisation for the Nvidia Tesla GPU architecture. OpenACC and CUDA commands were compiled using CUDA v10.0.130. All tools were linked against the Intel MPI library (v2018.4). I/O operations were executed via the sequential HDF5 library (v1.10.2) (Prabhat, 2014; The HDF Group, 1997-2020).

The analyses were executed on the TALOS computing cluster, a SUSE Linux Enterprise Server 15.1 SP1 system with 80 accessible computing nodes. Each node has two Intel Xeon Gold 6138 twenty-core processors with access to 188 GiB main memory in total. Furthermore, each node is equipped with two Nvidia Tesla Volta V100 (Nvidia Corporation, 2017) accelerator cards with 32 GB memory each.

The Message Passing Interface (MPI) library (Gropp *et al.*, 1998; Gropp *et al.*, 1999*b*; Gropp *et al.*, 1999*a*) was used to distribute ROIs across computing nodes at the coarse scale. At the finer scale, ROIs were delegated to Open Multi-Processing (OpenMP) (Chapman *et al.*, 2007) threads. The OpenMP threads were pinned to specific cores using OMP_PLACES=cores and executing one MPI process per node. In cases were the tools used the CPUs and GPUs, both GPUs of each node were utilised. Each GPU was instructed by one MPI process. Each MPI process spawned for this one OpenMP thread. All resources were used exclusively. The figures were generated using

Paraview (Ayachit, 2015) and Python. Analyses employed single precision, except for TetGen where double precision was employed.

Explicit calls to the MPI_Wtime and omp_get_wtime functions were made to monitor how much elapsed time the individual workflow steps took. I/O and non-I/O operations were distinguished. Two system variables were parsed on-the-fly to quantify virtual and resident main memory set sizes. These pieces of information were parsed from the /proc/self/stat system file.

1.5. Reconstruction of the experimental datasets

Aluminium bicrystal We used the following reconstruction parameters:

- Image compression factor ICF = 1.53,
- Field factor $k_f = 4.91$,
- Flight path length $\mathcal{L}_{fp} = 100\,\mathrm{mm}$,
- Detection efficiency $\eta = 0.8$.

For the real space method we computed $N_v = 40962$ SDMs per ROI with $m = 8$ binning. The signatures $\mathcal{S}_c^{msr}$ were composed by assigning each node the highest amplitude of the its corresponding amplitude spectrum in the bin interval corresponding to $\pm 1.823\,\text{Å}$ to $2.230\,\text{Å}$. Conceptually, this is equivalent to an indexing where one probes for all six crystallographic directions $\langle 1\,0\,0 \rangle$. In total $\hat{N}_v = 1000$ nodes were evaluated during indexing. Solutions up to the 1000-th closest candidates were computed for each N_g. Although reporting a single solution, like the one with the lowest image difference would be sufficient, we inspected such a large number of solutions to explore where the indexing fails and to understand this systematically.

Al-Li-Mg specimens The Al-Li-Mg-Ag dataset contained 45.17×10^6 ions of which a fraction of 4.96 % were ranged as lithium and 4.04 % as magnesium, respectively. Amplitude spectra to build signatures for the aluminium crystal structure candidate

were probed on the bin interval corresponding to ±1.823 Å to 2.223 Å lattice spacing. Amplitude spectra to build signatures for the Al_3Li crystal structure candidate were probed on the bin interval corresponding to ±1.805 Å to 2.206 Å lattice spacing. This corresponds to indexing based on the $\langle 2\,0\,0 \rangle$ plane stacks of the respective sub-lattices. The same indexing settings as for the aluminium bicrystal dataset were set but now compared to three instead of one reference pattern: one reference defined by a pure aluminium crystal and two for the aluminium and the lithium sub-lattices of a Al_3Li single crystal respectively.

2. Additional results

2.1. Robustness of the real space method against randomly missing atoms

Figure 2 reports how the signatures computed from the perfect single crystals change when randomly removing atoms from the lattice. The results show descriptive spatial statistics for all ROIs and SDMs. The very low spread of the quantile values documents that the signatures of every ROI have peaks of similar intensity (≈ 1.0). The background is in all cases at least ten times lower in intensity. The observation that the spread is similar for all η values substantiates a robustness against a random removal of atoms.

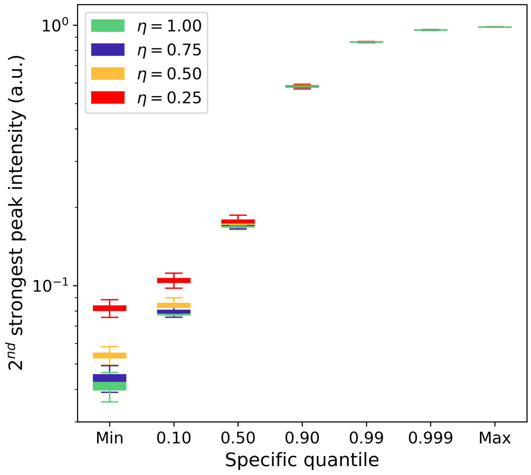

Fig. 2. Quantification of the real space method signal against noise due to missing atoms. For each ROI, we identified the peaks in all amplitude spectra and report the individually second strongest peak per amplitude spectrum. With N_v FE mesh nodes, i.e. N_v directions, this yields one cumulative distribution per ROI. Next, specific quantiles of the cumulative distribution are extracted for each ROI and displayed for the entire ROI ensemble. This condenses the statistics of how all amplitude spectra (40962 per ROI) for all ROIs (10000 in total) differ. We repeat this statistical analysis for all η values (the fraction of atoms remaining). The results show that the signal-to-noise ratio is not substantially affected by removing atoms.

2.2. Influence of the ROI radius and frequency resolution

Verification analyses on the same synthetic datasets but using larger radii for the ROIs did not improve the indexing quality for the noisy datasets. In fact, the exemplar SDM in Fig. 4 and corresponding amplitude spectra in Fig. 3 summarise that up-scaling the ROI only increases the total number of counts in the SDMs, i.e. the significance improves but the peaks get not better concentrated.

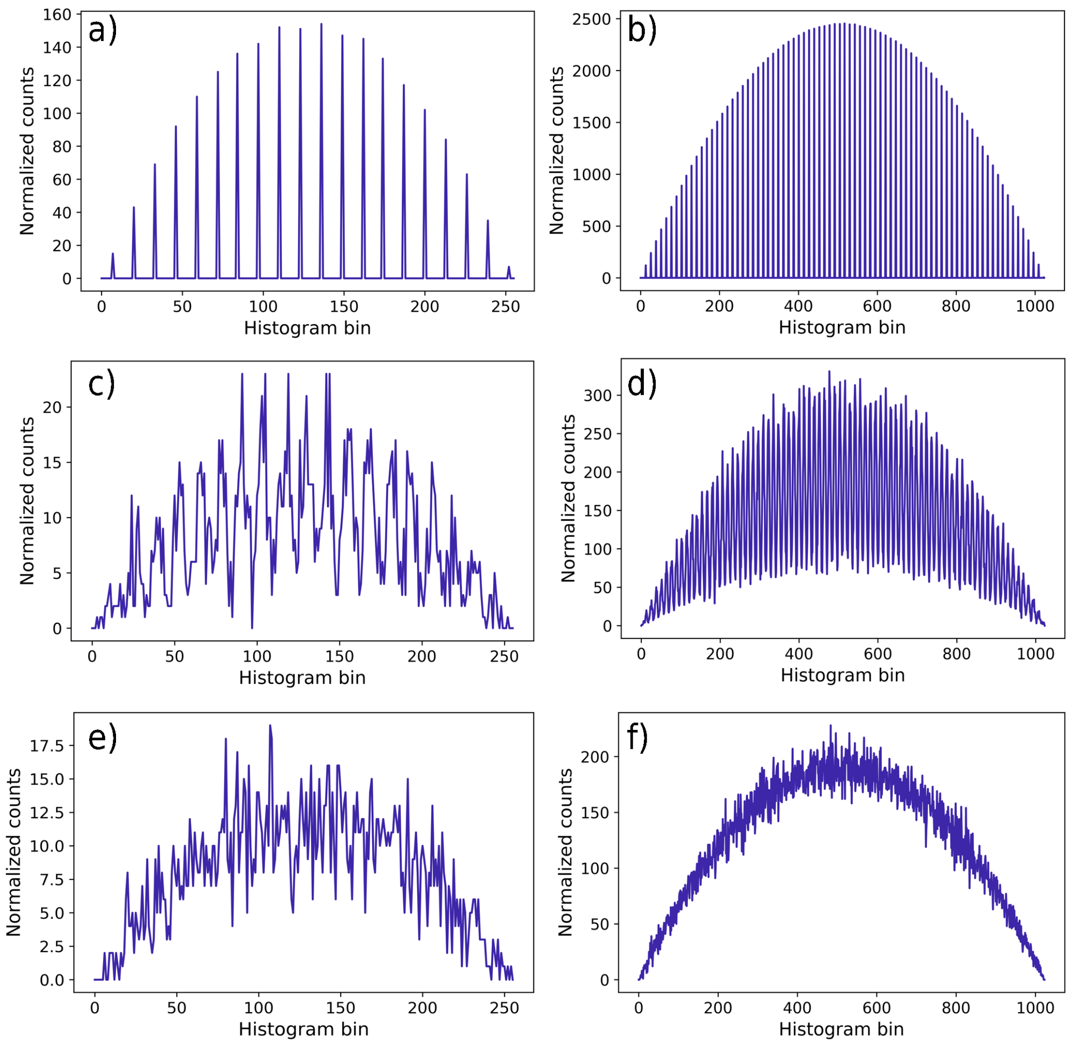

Fig. 3. Effect of up-scaling the radius of the ROIs for a fixed frequency resolution $\frac{2^{m-1}-1}{R} = 6.35\,1/\text{Å}$ and increasingly stronger positional noise. The left column a), c), e) displays exemplar SDMs for $R = 20\,\text{Å}$. The right column b), d), f) displays SDMs for the same projection direction in a), c), e) but a larger ROI radius $R = 80\,\text{Å}$, respectively. Up-scaling the ROI increases the significance of the histogram but does not better concentrate the peaks.

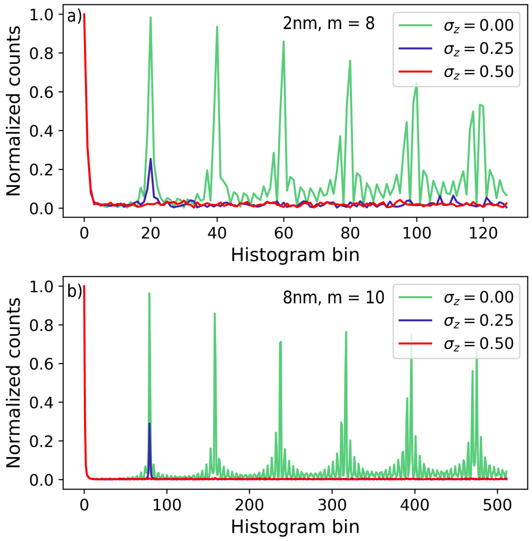

Fig. 4. The amplitude spectra for the above histograms support that an up-scaling of the ROI increases only the significance of the SDMs but does not better concentrate the peaks.

2.3. *Statistical analysis of the signatures for the aluminium bicrystal*

We summarise in the main paper that it is possible to detect regions in the datasets with significant crystallographic signal. Figure 5 documents the results of a successful protocol for performing a local refinement of the ROI grid. Figure 6 supports these results with a statistical analysis of the signal strength and the number of atoms per ROI.

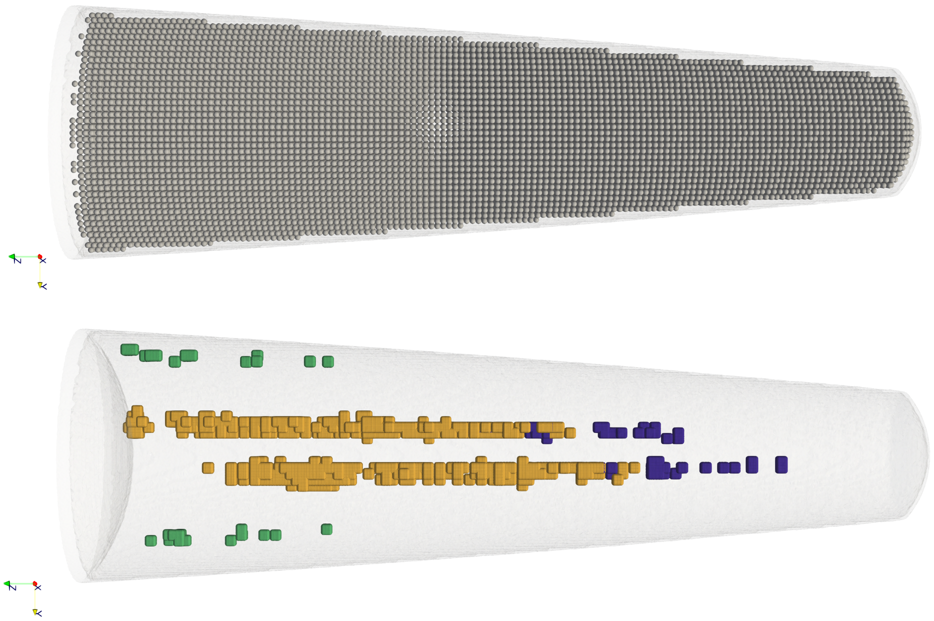

Fig. 5. The local ROI grid refinement allows to explore specifically those regions in the dataset more efficiently where the chance for successful indexing are best. The upper figure displays a coarse regular ROI grid (see explanations below). The lower figure displays signature-specific results for the environment of the refined ROIs. Different colours distinguish different signatures ($\{0\,0\,2\}$ in dark blue, $\{2\,2\,0\}$ in green, and $\{1\,1\,1\}$ in orange, respectively).

Such a local grid refinement works for example as follows: First, we scanned the dataset with a coarse resolution, here using $(20\,\text{Å})^3$. Approximately 1.0×10^5 ROIs were obtained as Fig. 5a) displays (with the ROIs rendered to scale as grey spheres). Intensities were evaluated individually for each ROI. Next, we filtered out, for each

pole ($\{h\,k\,l\}$) separately, those ROIs whose maximum image intensity was at least 0.75. Finally, we performed a grid refinement for these ROIs ($0.5\,\mathrm{nm}^3$). In effect, between 4.0×10^3 to 8.3×10^4 now more closely spaced ROIs were characterised. The dataset volume in Fig. 5b) shows that these ROIs probe different regions of the dataset. By contrast, a naive grid refinement of the coarse grid would have resulted in 64 times more computations, so our local refinement is at least one order of magnitude more efficient.

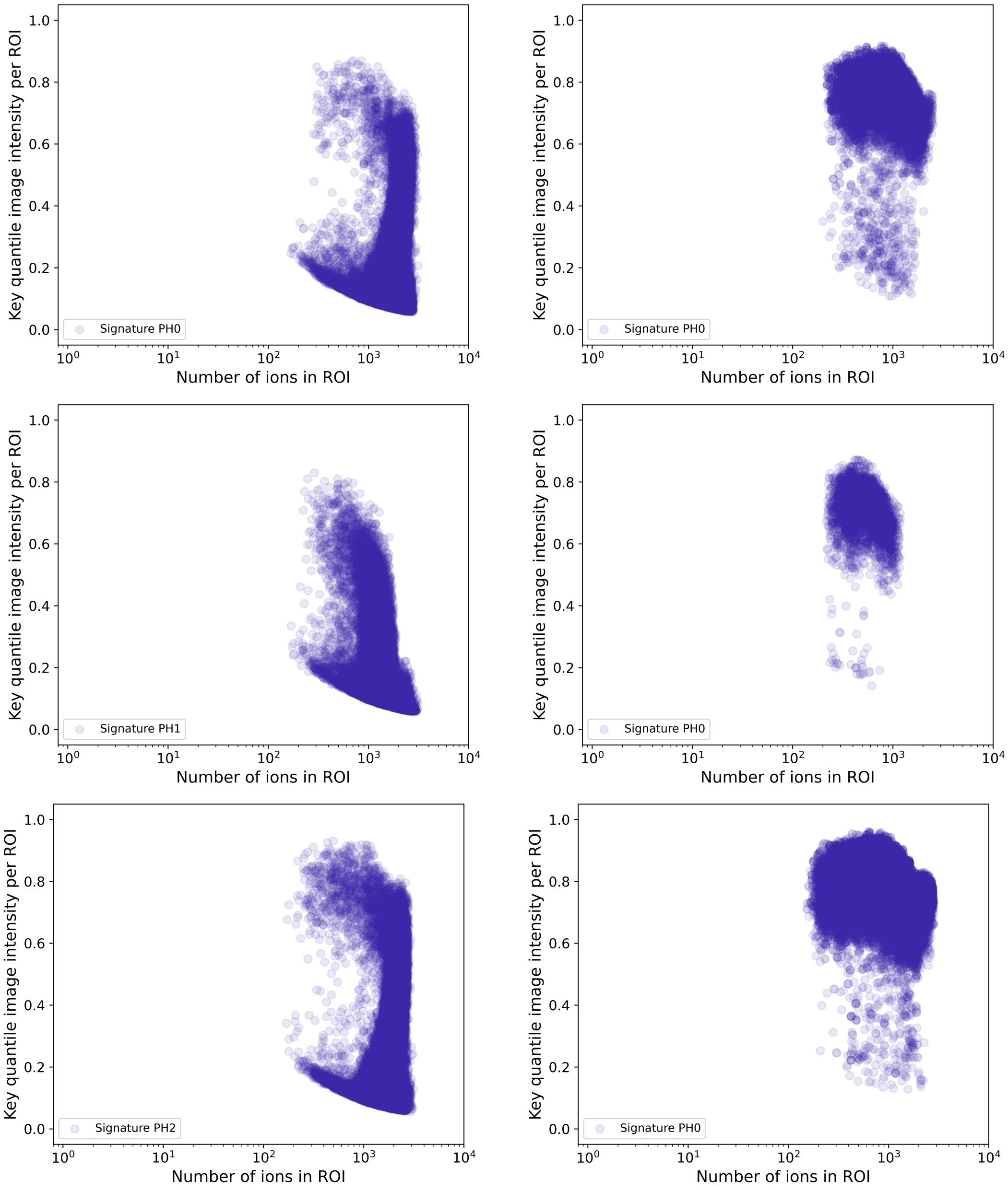

Fig. 6. Statistical analysis of the signal strength for three different poles $\{0\,0\,2\}$ (upper row), $\{2\,2\,0\}$ (middle row), and $\{1\,1\,1\}$ (lower row), respectively. We performed an analysis with an adaptive grid and compare how many atoms are included in a ROI and how high the signal intensities correlate with this atom count for each ROI. Specifically, we compare results for two ROI grids - a coarse one, or initial grid, respectively (left column) and a fine one, first refinement, respectively (right column). These results supplement and substantiate the findings to the aluminium bicrystal case study in the main paper. There are several regions in the dataset with strong crystallographic signal.

2.4. Statistical analysis of the signatures for the Al-Li-Mg-Ag dataset

We performed the above statistical analyses with grid refinement also for the Al-Li-Mg-Ag dataset. The same poles as the ones above were analysed but now two signatures computed for each $\{h\,k\,l\}$ - one signature for the aluminium and one for the lithium sub-lattice, respectively. Figure 7 shows the results, exemplified for $\{0\,0\,2\}$ signatures.

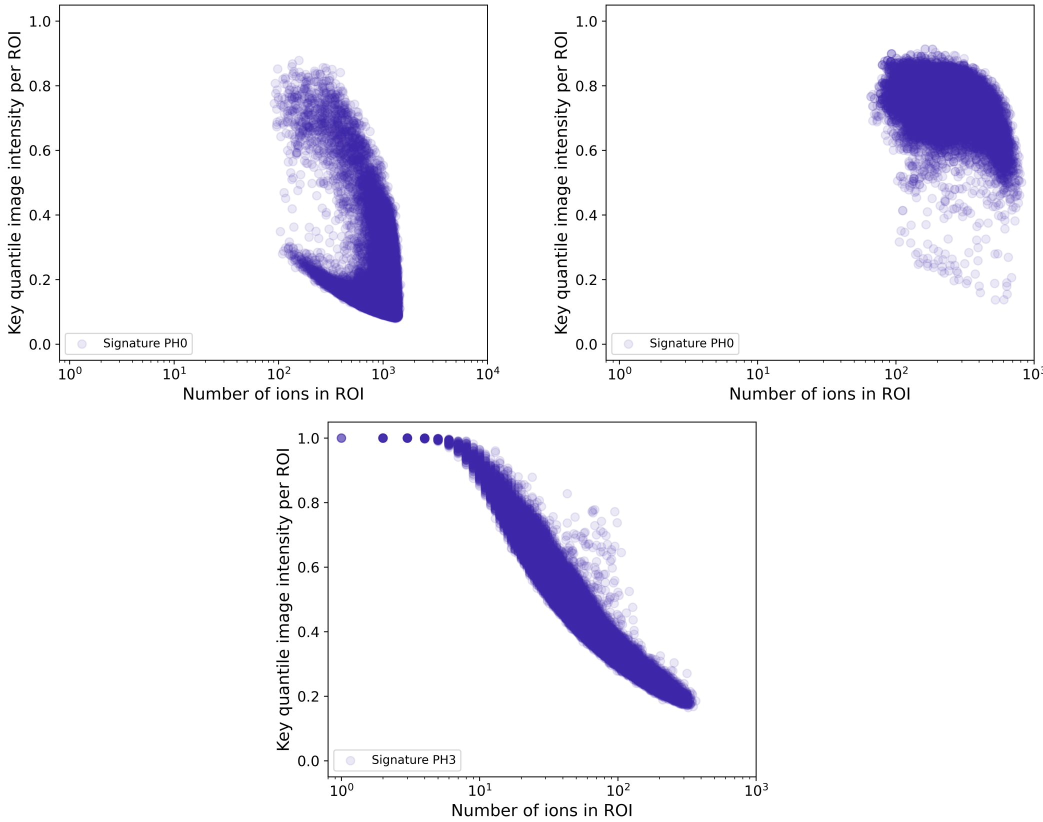

Fig. 7. Statistical analysis of the signal strength for $\{0\,0\,2\}$ signatures. The upper row shows statistics for aluminium atoms and compares the coarse (results to the left) with the fine grid (results to the right, using the same thresholding of image intensities like in Fig. 6). Below is the result for the coarse grid and $\{0\,0\,2\}$ signatures computed based on the lithium atoms. For further methodological details see text and explanation of the figure above. The results support the conclusion of the main paper that finite counting effects can result in strong peaks and have to be carefully separated from ROIs with more significant signal. The lower the number of atoms in a ROI is the noisier and eventually even skewed the SDMs become. When fast-Fourier-transforming these SDMs, this translates into high peaks in the amplitude spectra, i.e. high image intensities in the signatures. This is especially pronounced for ROIs which contain less than a few dozen lithium atoms.

2.5. Benchmarking: reasons for limited scalability

There are multiple reasons for limited scalability: 10000 ROIs cannot be equally distributed on 3200 CPUs cores. In effect, work load differences limit the scalability. In addition, there are a few code portions that remained sequentially executed. A detailed analysis of the elapsed time data showed that there are costs of synchronisation bottlenecks. These could be worth a future inspection and software optimisation. However, reducing these bottlenecks will require efforts and implementation techniques that are beyond the interest and toolkit of most atom probe practitioners and should therefore not be pursued here.

2.6. Preliminary analysis of compressing signatures with spherical harmonics

In what follows, we summarise the mathematical details of the discussed spherical harmonics compressing. First, we discretised a unit sphere into a geodesic sphere finite element mesh to allow for a mesh of near equal-area elements and a near constant density of nodal points per unit surface area. The spherical image (the signatures) to be fit is evaluated at the nodal points, resulting in a vector of values, $\{\epsilon\}$ (length n^n, the number of nodes on the geodesic mesh). A set of spherical harmonic modes are similarly defined at the nodal points of the geodesic mesh, given as $\left\{H^k\right\}$ (length n^n). With the spherical harmonic modes defined, the spherical image is described via a series expansion:

$$\{\epsilon\} = \sum_{k=1}^{n^h} w^k \left\{H^k\right\}, \tag{4}$$

where n^h is the number of harmonic modes in the series expansion. To solve for the weights, w^k, a method of least squares is used:

$$[H]^T [H] \{w\} = [H]^T \{\epsilon\} \tag{5}$$

Here, $\{w\}$ is a vector of the weights of the series expansion (length n^h), and $[H]$ is a matrix of dimension $n^n \times n^h$, constructed of nodal values of each harmonic mode:

$$[H] = \left[\left\{H^1\right\}\left\{H^2\right\}\left\{H^3\right\}...\left\{H^{n^h}\right\}\right] \tag{6}$$

The resulting vector of harmonic weights yields a reduced-order description of the spherical image. We discuss in the main paper that the spherical harmonics approach captures the location of the peaks. However, it smears out the intensity substantially. The QQ plot in Fig. 8 quantifies this visual impression.

a)

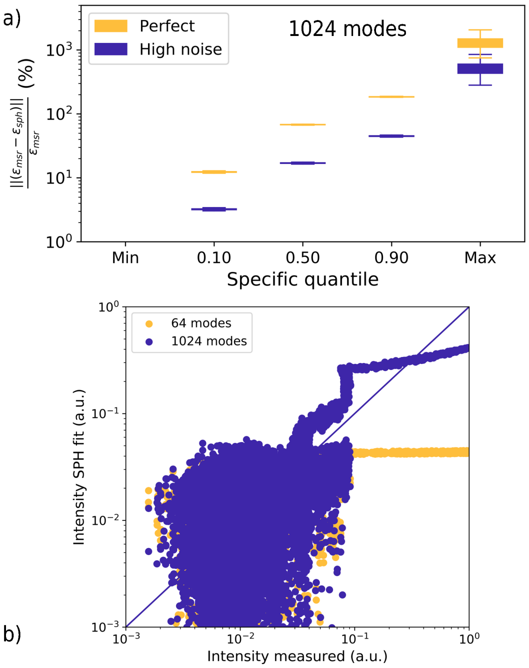

b)

Fig. 8. Approximating signatures of the aluminium single crystal synthetic datasets with a series of spherical harmonics. Peak positions are in principle captured but the intensities remain smeared out even for a very large number of modes.

References

Araullo-Peters, V. J., Gault, B., Shrestha, S. L., Yao, L., Moody, M. P., Ringer, S. P. & Cairney, J. M. (2012). *Scripta Materialia*, **66**, 907–910.

Ayachit, U. (2015). *ParaView: An End-User Tool for Large Data Visualization*. Kitware Inc., 1st ed.

Bachmann, F., Hielscher, R., Jupp, P. E., Pantleon, W., Schaeben, H. & Wegert, E. (2010*a*). *Journal of Applied Crystallography*, **43**, 1338–1355.

Bachmann, F., Hielscher, R. & Schaeben, H. (2010*b*). *Solid State Phenomena*, **160**, 63–68.

Bonnet, R. (1980). *Acta Crystallographica A*, **36**, 116–122.

Chapman, B., Yost, G. & van der Paas, R. (2007). *Using OpenMP: Portable Shared Memory Parallel Programming.* MIT Press, Cambridge, 1st ed.

Da, T. K. F., Loriot, S. & Yvinec, M. (2018). In *CGAL User and Reference Manual.* CGAL Editorial Board, 4.12 ed.
URL: *https://doc.cgal.org/4.12/Manual/packages.html#PkgAlphaShapes3Summary*

Frigo, M. & Johnson, S. G. (2005). *Proceedings of the IEEE*, **93**(2), 216–231.

Grimmer, H. (1974). *Acta Crystallographica*, **30**, 685–688.

Gropp, W., Huss-Lederman, S., Lumsdaine, A., Lusk, E., Nitzberg, B., Saphir, W. & Snir, M. (1998). *MPI - The Complete Reference, Volume 2, The MPI Extensions.* MIT Press, Cambridge, 1st ed.

Gropp, W., Lusk, E. & Skjellum, A. (1999*a*). *Using MPI-2 Advanced Features of the Message Passing Interface.* MIT Press, Cambridge, 1st ed.

Gropp, W., Lusk, E. & Skjellum, A. (1999*b*). *Using MPI: Portable Parallel Programming with the Message Passing Interface.* MIT Press, Cambridge, 2nd ed.

Heinz, A. & Neumann, P. (1991). *Acta Crystallographica A*, **47**, 780–789.

Hennessy, J. L. & Patterson, D. A. (2012). *Computer Architectures: A Quantitative Approach.* Morgan Kaufmann, Amsterdam, 5th ed.

Intel, (2019). Intel Parallel Studio XE 2019.
URL: *https://software.intel.com/en-us/articles/intel-c-compiler-190-for-linux-release-notes-for-intel-parallel-studio-xe-2019*

Jeffers, J. & Reinders, J. (2015). *High Performance Parallelism Pearls Volume Two: Multicore and Many-Core Programming Approaches.* Morgan Kaufmann.

Kaiser, J. F. & Schafer, R. W. (1980). *IEEE Transactions on Acoustics, Speech, and Signal Processing*, **28**(1), 105–107.

Kühbach, M., (2020). PARAPROBE — Strong-Scaling Tools for Open-Source Mining of Atom Probe Tomography Data — Source Code.
URL: *https://gitlab.mpcdf.mpg.de/mpie-aptfim-toolbox/paraprobe.git*

Kühbach, M., Bajaj, P., Zhao, H., Çelik, M. H., Jägle, E. A. & Gault, B. (2021). *npj Computational Materials*, **7**(21).

Morawiec, A. (2004). *Orientations and Rotations.* Springer, Berlin, 1st ed.

Nvidia Corporation, (2017). Nvidia Tesla V100 GPU Architecture.
URL: *https://images.nvidia.com/content/volta-architecture/pdf/volta-architecture-whitepaper.pdf*

Nvidia Corporation, (2019). CUDA Toolkit Documentation.
URL: *https://docs.nvidia.com/cuda*

Popko, E. S. (2012). *Divided Spheres: Geodesics and the Orderly Subdivision of the Sphere.* CRC Press, Boca Raton, 1st ed.

Portland Group Inc., (2020). Documentation to the PGI 19.10 compiler.
URL: *http://www.pgroup.com/resources/docs/19.10/x86/index.htm*

Prabhat, Q. K. (ed.) (2014). *High Performance Parallel I/O.* Chapman & Hall, CRC Computational Science, Boca Raton, 1st ed.

Prabhu, K. (2013). *Window Functions and Their Applications in Signal Processing.* Boca Raton: Taylor & Francis Group, 1st ed.

Rauber, T. & Rünger, G. (2013). *Parallel Programming for Multicore and Cluster Systems.* Springer, Heidelberg, 2nd ed.

Reinders, J. & Jeffers, J. (2014). *High Performance Parallelism Pearls Volume One: Multicore and Many-Core Programming Approaches.* Morgan Kaufmann, 1st ed.

Reinhard, D. A., Payne, T. R., Strennen, E. M., Oltman, E., Geiser, B. P., Sobering, G. S. & Mandt, J. (2019). *Microscopy and Microanalysis*, **25**, 302–303.

Schäling, B. (2014). *The Boost C++ Libraries*. XML Press, 2nd ed.

Si, H. (2015). *ACM Transactions on Mathematical Software*, **41**(2), 1–36.

Strobl, S., Formella, A. & Pöschel, T. (2018). *Journal of Computational Physics*, **311**, 158–172.

The CGAL Project (2018). *CGAL User and Reference Manual*. CGAL Editorial Board, 4th ed.
URL: *https://doc.cgal.org/4.12/Manual/packages.html*

The HDF Group, (1997-2020). Hierarchical Data Format Version 5 (HDF5). Http://www.hdfgroup.org/HDF5/.

Ulfig, R. M., Prosa, T. J., Chen, Y., Rice, K. P., Martin, I., Reinhard, D. A., Geiser, B. P., Oltman, E., Lenz, D. R., Bunton, J., van Dyke, M., Kelly, T. F. & Larson, D. J. (2017). *Microscopy and Microanalysis*, **23**(Suppl 1), 40–41.

Vurpillot, F., Da Costa, G., Menand, A. & Blavette, D. (2001). *Journal of Microscopy*, **203**(3), 295–302.
